# Gene-edited murine cell lines for propagation of chronic wasting disease prions

**DOI:** 10.1038/s41598-019-47629-z

**Published:** 2019-08-01

**Authors:** Rupali Walia, Cheng Ching Ho, Chi Lee, Sabine Gilch, Hermann M. Schatzl

**Affiliations:** 1 Department of Comparative Biology & Experimental Medicine, Faculty of Veterinary Medicine, University of Calgary, Calgary, Alberta T2N 4Z6 Canada; 2 Calgary Prion Research Unit, University of Calgary, Calgary, Alberta T2N 4Z6 Canada

**Keywords:** Neurodegeneration, Prion diseases

## Abstract

Prions cause fatal infectious neurodegenerative diseases in humans and animals. Cell culture models are essential for studying the molecular biology of prion propagation. Defining such culture models is mostly a random process, includes extensive subcloning, and for many prion diseases few or no models exist. One example is chronic wasting disease (CWD), a highly contagious prion disease of cervids. To extend the range of cell models propagating CWD prions, we gene-edited mouse cell lines known to efficiently propagate murine prions. Endogenous prion protein (PrP) was ablated in CAD5 and MEF cells, using CRISPR-Cas9 editing. PrP knock-out cells were reconstituted with mouse, bank vole and cervid PrP genes by lentiviral transduction. Reconstituted cells expressing mouse PrP provided proof-of-concept for re-established prion infection. Bank voles are considered universal receptors for prions from a variety of species. Bank vole PrP reconstituted cells propagated mouse prions and cervid prions, even without subcloning for highly susceptible cells. Cells reconstituted with cervid PrP and infected with CWD prions tested positive in prion conversion assay, whereas non-reconstituted cells were negative. This novel cell culture platform which is easily adjustable and allows testing of polymorphic alleles will provide important new insights into the biology of CWD prions.

## Introduction

Prion diseases, such as bovine spongiform encephalopathy (BSE), chronic wasting disease (CWD) in cervids, and Creutzfeldt-Jakob disease (CJD) in humans, are a family of fatal neurodegenerative diseases caused by the conversion of the cellular prion protein (PrP^C^) to the abnormally folded isoform (PrP^Sc^)^1–4^. An increasing number of CWD cases has been reported across North America, South Korea and also recently in Europe^5–7^. CWD negatively impacts cervid populations and may have zoonotic potential, as did BSE/mad cow disease^8–11^.

Cell culture models are important for studying crucial aspects of prion propagation and transmission between species. Although robust animal models are available, many of them are limited to propagating prions of a single species or certain strains of prions. Cellular platforms which complement these animal models are either unavailable for a large number of prions or propagate them transiently^12–14^. Traditionally cell lines were screened empirically for susceptibility, with accumulation of newly propagated PK-resistant PrP detected by immunoblot analysis as the main read-out for successful infection. Subsequent single cell cloning of infected cells or pre-selection of clones was a critical step in establishing highly susceptible cell lines^15^. Since the development of these cell lines relied on the innate property of the cell line for propagating prions, they remained constrained by their genetic makeup and hence the permissiveness of the cell line towards prion infection. The most widely used infection models are the mouse neuronal cell lines like N2a and its subclones and GT1^16–19^. Mouse fibroblasts and rabbit kidney cells (RK13) stably expressing exogenous PrP are some of the non-neuronal cell lines that have been used for infection studies^20,21^. PrP-reconstituted RK13 cells can propagate rodent, sheep and cervid prions, whereas N2a, fibroblast and GT1 cells can only propagate mouse-adapted prions^22^.

Most of the cell models developed so far have a fairly narrow susceptibility range to prion strains, making cross species comparisons difficult. To develop protean cellular platforms, capable of investigating new and emerging prion diseases like CWD, we propose to use genome engineering of susceptible cell lines to expand their repertoire of susceptibility and ability to propagate prions. The presentation of native PrP^c^ on the cell surface and the subsequent conformational changes in endocytic compartments which convert this substrate into the scrapie isoform determine the susceptibility of a cell line^13–16^. This aspect would be difficult to engineer since it involves a complex interplay of multiple factors many of which are unknown^23^. However, the causes behind the narrow infection range could simply be due to the mismatch between donor PrP^Sc^ template and recipient PrP^c^ substrate. In that case, increasing the infection range would require the simpler task of replacing the native substrate with a matching transgenic or even universal substrate to create cellular infection models accepting a wider range of prions. These novel cellular models would significantly contribute to our understanding of the role of prion protein polymorphisms on prion propagation.

CRISPR- Cas9 tools (CC9) are now readily available and can be exploited for precise genome editing^24–26^. CC9 has been successfully used to manipulate the native prion protein gene in a number of cell lines like N2a, C2C12 myocytes and NMuMG^27,28^. In the present work, we knocked-out the endogenous murine PrP and reconstituted the cells with a more promiscuous bank vole PrP or specific cervid PrP alleles. While the ablation of the endogenous PrP expression eliminated background interference, the introduction of these transgenic PrP substrates made the cells permissive to CWD prion propagation. Two murine cells lines were used in this study: CAD5, a highly susceptible subclone derived from a CNS catecholaminergic cell line (Cath.a/CAD), and immortalized embryonic fibroblasts (uncloned MEF cells) representing a non-neuronal background^29–31^. The targeted deletion of endogenous PrP using paired gRNAs resulted in a loss of PrP expression and resistance to infection with mouse prions. CAD5 and MEF knockout cells were reconstituted with bank vole (BV) PrP and successfully infected with rodent prions and cervid prions, isolated from white-tailed deer (WTD) and mule deer (MD). This represents a significant increase in the species range of these cells for prions while providing for the first time gene-edited mouse cell lines which have the potential to propagate cervid prions. We also reconstituted MEF-KO cells with cervid PrP and obtained a positive infection phenotype when challenged with cervid prions, even without enriching for highly susceptible cells.

In summary, we provide a successful strategy for designing cell lines with specific as well as broader susceptibility to prion infection, while retaining a common host background. Stable infection can be achieved by future enrichment for highly susceptible and persistently infected cells via subcloning. Data obtained by infecting such gene-targeted and re-constituted cultured cells will provide important new insights into species and prion strain barriers and provide new experimental systems for studying prions *in vitro*.

## Results

### Generation and enrichment of PrP knock out cells using a dual-gRNA strategy coupled with FACS enrichment

**Figure 1:**
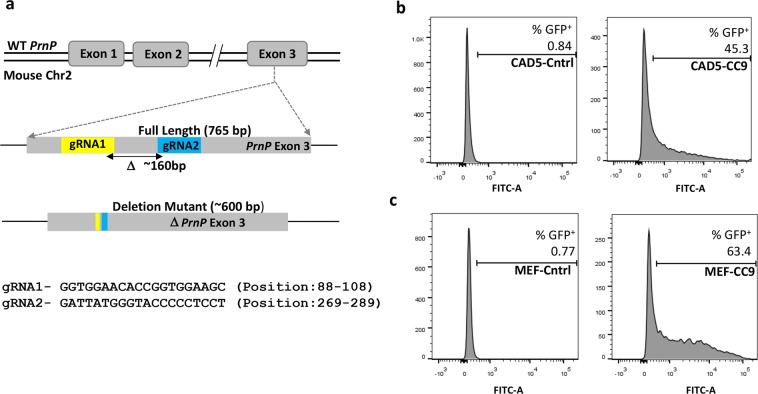
Targeted deletion of *Prnp* exon 3 using paired gRNA’s and FACS enrichment of edited cells. (**a**) Schematic representation of locations of the two guide RNAs (gRNA1 and gRNA2) targeting the exon 3 locus of the mouse *Prnp* gene (765 bp). gRNA1 (yellow) and gRNA2 (blue) are located ~160 bp apart and result in a deletion mutant of approximately 600 bp. (**b**) Flow cytometric enrichment of targeted cells. Left panel shows non-transfected CAD5 cells (control). Right panel shows CAD5 cells (CAD5-CC9) co-transfected with two plasmids carrying the gRNA’s (pX458-gRNA1 & pX459-gRNA2) showing 45% GFP positive cells. (**c**) Left panel shows non-nucleofected MEF cells (control). Right panel shows MEF cells (MEF-CC9) co-nucleofected with pX458-gRNA1 & pX459-gRNA2 showing 63% GFP positive cells. The GFP^+^ cells were subsequently sorted and expanded into single cell clones.

Since the endogenous mouse PrP^c^ may serve as a template for the PrP^Sc^ inoculum and/or create interference during infection studies with non-mouse prions, it was essential to first remove the host cellular *Prnp* gene^23^. A CRISPR–Cas9 strategy was used with dual-gRNAs targeting opposite strands of the exon 3 *Prnp* locus to facilitate larger deletions of the *Prnp* gene. This would allow a quick PCR-based identification of the knock out cells. The efficiency of generating on-target mutations has also been reported to be much higher with the use of a pair of gRNAs targeting opposite strands of the same gene, rather than a single gRNA^32,33^. The guide RNAs were designed to target the *Prnp* coding sequence within exon 3 since it has been shown to encode the entire PrP open reading frame^34^. The selected sites, approximately 160 bp apart (Fig. 1a), were predicted by the ‘CRISPR Design Tool’ (http://crispr.mit.edu) in order to ensure a minimum number of off-target sites in the mouse genome^35^. The gRNA1 and gRNA2 were cloned at the *BbsI* site into the CRISPR plasmids pX458 (Addgene plasmid # 48138) and pX459 (Addgene plasmid # 48139) having GFP and puromycin as selection markers, respectively. The host cells were simultaneously co-transfected with both these plasmids but screened only for GFP expression. The puromycin resistance phenotype requires longer incubation periods and often does not show up in transiently transfected cells. Thus, successfully co-transfected cells having the desired deletions may not exhibit the resistant phenotype and we could end up losing potential knockouts in the screening process. Lipofectamine-based delivery of these gRNA plasmids in CAD5 cells resulted in 45% GFP positive cells (Fig. 1b). This showed that the ‘transfectability’ of the cells was reasonably high and we could infer that a significant fraction of cells would have received both plasmids. Therefore, we relied on GFP based FACS sorting to screen only for pX458 uptake, assuming that a significant fraction of these “single transfected” cells would also contain the second plasmid. This strategy resulted in the indirect enrichment of co-transfected cells having both gRNA1 and gRNA2 donor backbones. MEF cells on the other hand are more resilient to transfection reagents and hence required nucleofection for efficient delivery of CRISPR reagents into this cell line^36^. Using optimized conditions we obtained 63% GFP positive MEF cells post nucleofection (Fig. 1c).

48 hours post transfection/nucleofection cells were sorted into 96-well plates such that a single cell was plated into each well to ensure clonal isolation. Since MEFs are larger in size FACS sorting was optimised using a wider sort nozzle (130 μm) which increased the viability of sorted cells significantly. The clonal cell expansions were visually monitored for about two weeks and then processed for characterising the knock-out phenotype.

### Characterization of *indels* in PrP-KO cells by amplicon analysis

**Figure 2:**
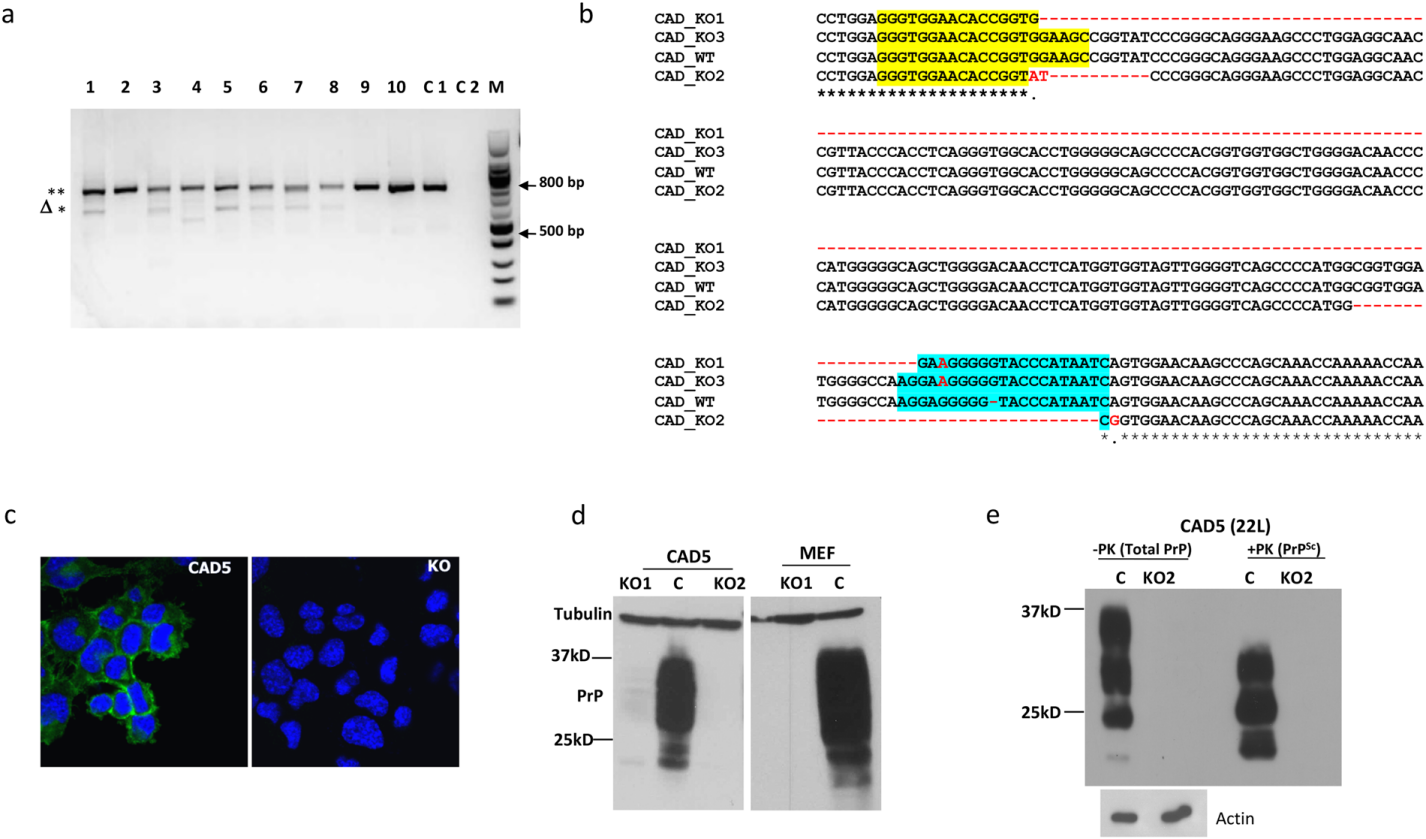
Characterization of PrP-KO cells. (**a**) Genomic PCR from clonal isolates of CAD5-KO cells using PrP-*F* and PrP-*R* primers to amplify the complete 765 bp *Prnp* exon3. Lanes 1–10 represent PCR signature from single cell clones: the upper 765 bp (**) band represents the full-length exon 3 while the lower bands (Δ*) represent large deletions. Lane C1 represents the genomic PCR from the un-transfected CAD5 cells (control) and C2 represents the non-template negative control. M is the 100 bp molecular weight marker. (**b**) Sequence comparison of PCR amplicons (KO1, KO2 and KO3) from a representative single cell CAD5 knock out clone with original wild type (CAD_WT) demonstrating a range of *indels* between the two target sites. Yellow and blue regions represent the gRNA1 and gRNA2 sites, respectively. Large deletion in KO1 is represented by dotted line in red. Sequences in red represent *indels*. (**c**) Representative confocal microscopy image of parental CAD5 (left panel) and CAD5-KO2 cells (right panel) showing absence of PrP signal in KO cells. mAb 4H11 was used as primary and Alexa fluor 488 goat anti-mouse as secondary antibody. Nuclei were stained with DAPI. (**d**,**e**) Immunoblot analysis of single cell clones of CAD5 (KO1, KO2) and MEF cells (KO1) using mAb 4H11 showing absence of PrP expression. Lane C represents PrP expression of parental cells. Tubulin was used as a loading control. (**f**) Immunoblot analysis demonstrating prion propagation resistant phenotype of CAD5-KO2. Control CAD5 and KO2 cells were infected with 1% brain homogenate of terminally ill 22L-infected mice and cell lysates were subjected to PK digestion to distinguish between total PrP and PrP^Sc^. (−PK) represents the total PrP and (+PK) represents PrP^Sc^. The blots were probed with anti-PrP mAb 4H11 and actin was used as a loading control.

Since we expected a large deletion between the two gRNA target sites, PCR primers were designed at the two ends of PrP exon 3 (Supplementary Table 1). This allows a quick identification of deletion mutants by observing a reduction in the size of the PCR amplicon (compared to enzyme-based mismatch cleavage assay). Genomic DNA (gDNA) from multiple clones was used as template to amplify the target region. The expected size reduction was observed in a large number of CAD5 clones (Fig. 2a). Interestingly, the PCR signature of these CAD5 clones also contained the full-length product, implying the presence of multiple copies of the *Prnp* gene in the host cells. Karyotyping of CAD5 cells was subsequently done to confirm that these cells indeed possess multiple chromosomes. To study the nature of these mutations, four single cell clones were selected and their PCR products were cloned into pCR ^TM^ BluntII-TOPO plasmids. These constructs were transformed into Stbl3 competent *E*. *coli* to obtain four amplicon mini-libraries per cell line. Plasmid inserts from 10–12 bacterial colonies from each of these libraries were sequenced and analyzed for the presence of *indels*. As expected, each set contained a dominant set of sequences with precise deletions of approximately 160 bp, representing the region flanked by the two gRNA sites. More interestingly, the other sequences showed variability in the *indels* at both gRNA target sites leading to premature stops and inactive PrP (Fig. 2b). Clearly, the gRNAs had targeted the multiple PrP copies present in CAD5 cells but modified each copy differently in the tested cell clones. On analysis we also found that the mini library from CAD5 KO1 had a sequence matching the wildtype allele which could possibly lead to low level residual PrP expression. Unlike this the mini library from CAD5 KO2 had disruptions/indels in all the sequences which prompted us to compare both these clones for efficient ablation of PrP expression (Fig. S1a). On the other hand, the amplicon sequence signature of the MEF clones predominantly showed only two types of *indels* leading to premature stop codons and frameshift mutations (Fig. S1b). To confirm the loss of expression, immunofluorescence staining was done with anti-PrP mAb 4H11 which showed a clear absence of PrP expression in the CAD5 KO2 cells as compared to the parental CAD5 cells (Fig. 2c). To test the possibility of any unedited copy of the *Prnp* gene remaining inside the cell leading to residual expression, we also performed immunoblotting. We observed a faint residual PrP signature in CAD5 KO1 cells while there was a complete loss of the PrP expression (PrP^−/−^) in CAD5 KO2 cells (Fig. 2d). Similarly, the MEF KO1 cells showed a complete loss of PrP expression (Fig. 2e).

In summary, these data show that our CRISPR gene editing approach successfully abolished PrP expression in CAD5 KO2 and MEF KO1 cells and hence these knockout cells were selected for all subsequent studies.

### Stable reconstitution of PrP^−/−^ cells using lentiviral transduction

The presence of residual PrP expression in KO cells may interfere with transgenic PrP studies. To confirm functional absence, CAD5 KO2 cells were infected with 22 L prions and monitored over several passages. Whereas non-edited control cells propagated PrP^Sc^, no infection was observed in KO2 cells, demonstrating the complete functional knockout of the PrP gene (Fig. 2f). A similar resistant phenotype was also obtained in MEF KO1 cells (*data not shown*). These PrP^−/−^ cells could now be reconstituted with exogenous or transgenic PrP, and serve as a cellular platform for infection studies. Lentiviral transduction was used to ensure stable integration of the heterologous *Prnp* gene. Reconstitution was first tested in the selected CAD5 and MEF knock out cells using the murine *Prnp* gene to confirm the ability of KO cells to regain their prion propagation phenotype. The coding region of mouse PrP (MoPrP) was cloned in a pCDH-dual promoter lentiviral vector containing both GFP and puromycin as selection markers. The transduced cells were selected on puromycin over multiple passages at the end of which 100% cells were found to be GFP positive by fluorescence microscopy. Stable integration and expression of the target gene in these cells was further confirmed by Western blot analysis (Fig. S2).

**Figure 3:**
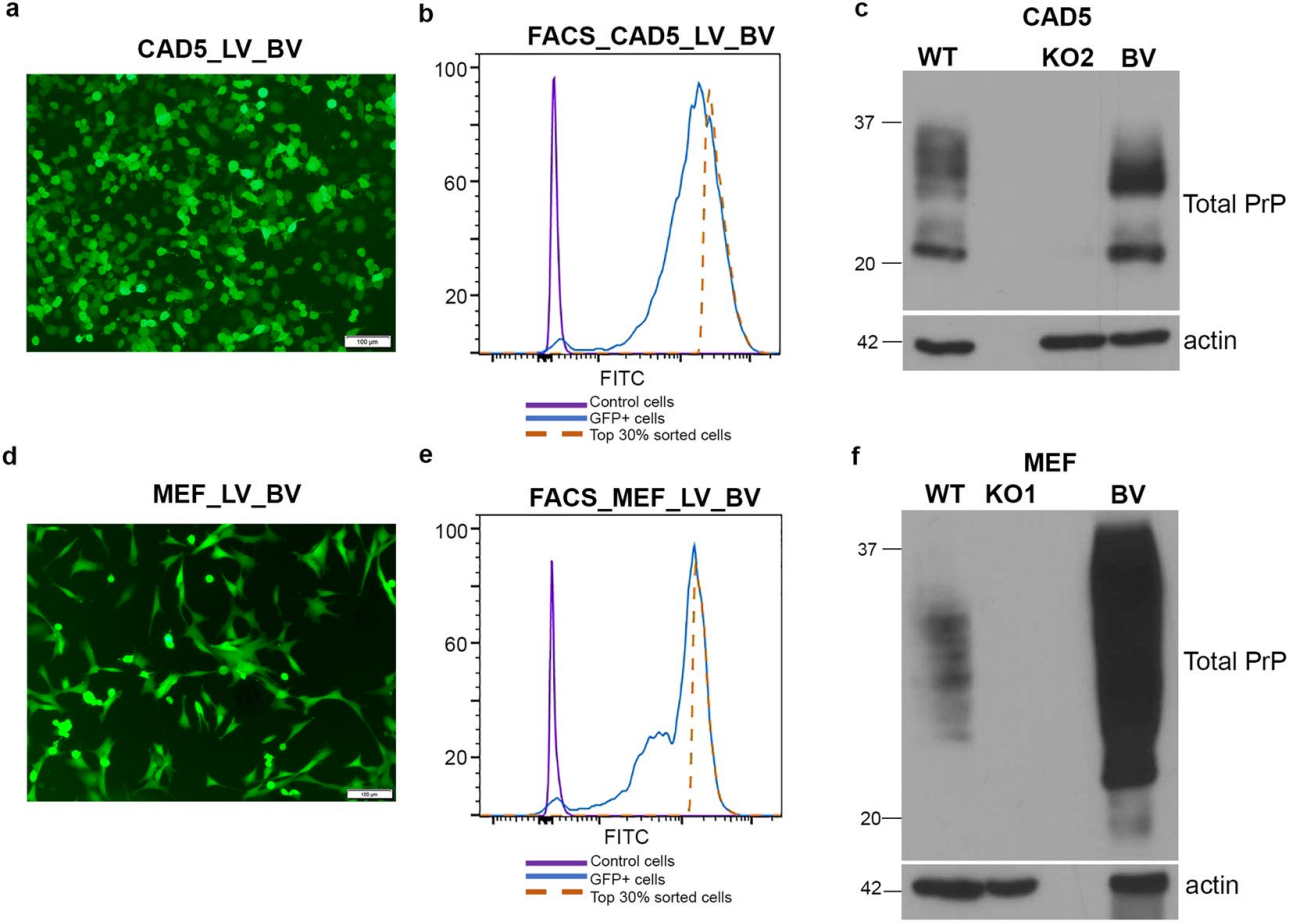
Stable reconstitution of PrP^−/−^ cells with bank vole PrP (BV-PrP) using lentiviral transduction. (**a**,**d**) Immunofluorescence imaging of CAD5 and MEF reconstituted cells. Recombinant lentiviruses expressing bank vole PrP were generated using pWP1-BV plasmid which simultaneously expresses BV-PrP and EGFP. PrP^−/−^ cells were transduced with these lentiviruses (CAD5_LV_BV and MEF_LV_BV) and the GFP^+^ signature was visualized with Olympus IX51 fluorescence microscope. (**b**,**e**) Enrichment of CAD5 and MEF transduced cells by sorting top 30% GFP^+^ hyper-expressors using FACS Aria cell sorter. The sorted cells were passaged and expanded into BV-PrP expressing stable cells. (**c**,**f**) Western blot showing stable integration and expression of bankvole PrP in CAD5 and MEF cells. WT represents the parental cells expressing mouse PrP and KO represents the PrP^−/−^ cells. BV represents expression of bank vole PrP in the sorted and expanded cell population. The blots were probed with anti-PrP mAb 4H11 and actin was used as loading control.

Simultaneously we decided to reconstitute CAD5 KO2 and MEF KO1 (PrP^−/−^) cells with the bank vole *Prnp* gene, since bank voles (BV) and transgenic mice expressing bank vole prion protein (BV-PrP) are known to be susceptible to most types of prions^37,38^. This would serve our ultimate goal of designing a universal infection model, for a range of prions. Since we were unable to get transductants using the pCDH construct, we changed the lentiviral backbone to pWP1 (Addgene#12254) which is a bicistronic vector that allowed simultaneous expression of BV-PrP and EGFP (pWP1-BV) under the EF-1α promoter (Fig. 3a,d). The IRES element placed between the two genes acts as another ribosome recruitment site, thereby resulting in co-expression of both proteins from a single mRNA. This allowed us to introduce a FACS-based enrichment step where cells having the highest levels of GFP were sorted. This provided us with a sub-population of transductants having a considerably higher likelihood of enhanced BV-PrP expression (Fig. 3b,e). These cells were passaged for several generations and stable integration of the BV-*Prnp* gene and its expression was confirmed by Western blot analysis (Fig. 3c,f). The pWP1 based cloning and FACS enrichment strategy was extended to express different cervid PrP alleles, WTD wild-type, 116AG and 138 N in both CAD5 KO2 and MEF KO1 cells. Stable integration was confirmed by immunoblot analysis (Figs S2 and S3). This clearly demonstrated that stable expression of different cervid PrPs (WTD, 116 G, 138 N) could be obtained with these engineered host backgrounds. These PrP polymorphisms or allelic variants in cervid *Prnp* are important determinants of susceptibility and pathogenesis^39–41^. Thus, both KO cell lines could be reconstituted with a range of transgenic/polymorphic variants vastly increasing the repertoire of cellular platforms available for quantitative *in vitro* analysis of prion strains, titers and biological properties using real time quaking-induced prion conversion (RT-QuIC) or standard scrapie cell assay (SSCA)^42,43^.

Taken together, lentiviral transduction allowed stable expression of a variety of PrP alleles in CAD5 and MEF knock out cells.

### Mo-PrP and BV-PrP expression in knock-out cells re-establishes prion infection by 22 L prions

**Figure 4:**
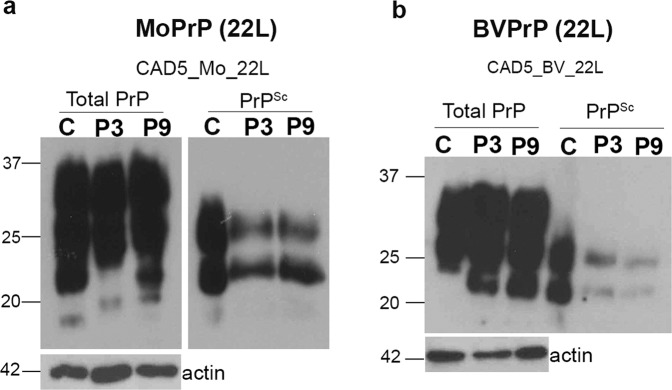
Stable infection of CAD5 cells reconstituted with Mo-PrP and BV-PrP by 22 L prions. (**a**) Immunoblot analysis demonstrating sustained infection phenotype of reconstituted MoPrP cells. Control CAD5 cells (C) and cells reconstituted with mouse PrP (CAD5_Mo) were infected with 1% brain homogenate from terminal ill mice infected with 22 L prions (CAD5_Mo_22 L). Cell lysates from passage P3 of parental CAD5 and passages P3 and P9 of the CAD5_Mo_22L cells were treated with and without proteinase K. The non-treated (−PK) represents the total PrP (left panel) while the treated lysate (+PK) represents PrP^Sc^ (right panel). The reconstituted CAD5-Mo cells show presence of PrP^Sc^ from passage P3 to P9 post-infection, demonstrating successful and persistent infection. (**b**) Immunoblot analysis demonstrating sustained infection of CAD5 KO cells reconstituted with bank vole PrP (CAD5_BV). Control CAD5 (C) and the CAD5_BV cells were incubated with 1% brain homogenate from mice infected with 22L prions (CAD5_BV_22L) and processed as above. The reconstituted CAD5_BV cells showed presence of PrP^Sc^ from passage P3 to P9 post-infection (right panel), demonstrating successful and persistent infection.

PrP^C^ expression has been shown to play a primary role in prion infection and disease pathology. We therefore expected that the reconstituted CAD5 KO2 cells expressing either mouse (CAD5_Mo) or bank vole PrP (CAD5_BV) would be rendered permissive to mouse adapted prions. To this end, cells were infected with 1% of brain homogenate from terminally ill 22L- infected mice and tested for the presence of PrP^Sc^ by immunoblot analysis. A strong PrP^Sc^ signal was observed at passage P3 and P9 in CAD5_Mo_22L cells, clearly demonstrating the recovery of the infection phenotype by complementation in these cells (Fig. 4a). A weaker PrP^Sc^ signal was also observed at passages P3 and P9 of CAD5_BV_22L cells (Fig. 4b). These results confirmed that the stable expression of heterologous PrP in the reconstituted knock-out cells was sufficient to allow PrP^Sc^ propagation even after nine passages post-infection, without enrichment for highly susceptible cells. Similar results were obtained with reconstituted MEF cells when tested for 22L prion infection.

In summary, PrP reconstituted cells regained their ability to propagate mouse 22L prions, when expressing mouse or bank vole PrP as substrate.

### Demonstrating the versatile nature of engineered murine cells to propagate cervid prions

**Figure 5:**
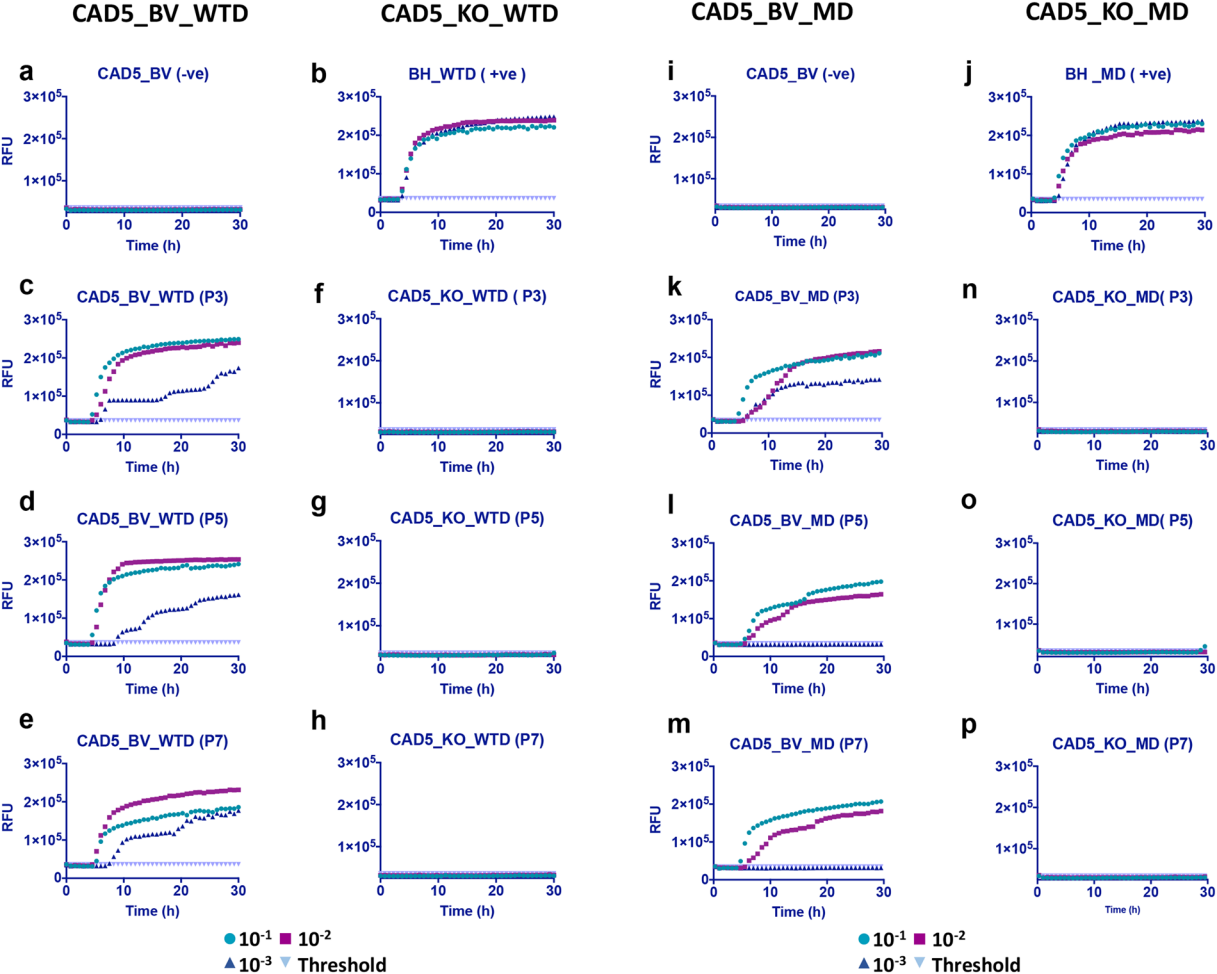
RT-QuIC analysis showing positive prion seeding activity in CAD5_BV cells infected with white-tailed deer (WTD) and mule deer (MD) prions. Post infection cell lysates were collected at passages P3, P5, P7 and subjected to NaPTA precipitation and ten-fold concentration. Recombinant mouse PrP was used as substrate. Each RT-QuIC reaction was seeded with 2 μl of concentrated cell lysate in quadruplicate and at three dilutions (10^−1^ to 10^−3^). The average increase of Thioflavin-T fluorescence in replicate wells (RFU) is plotted as a function of time (hours). Each panel shows fluorescence profiles for all three dilutions (10^−1^ to 10^−3^) and the threshold cut-off values. A positive seeding activity was seen at passage P3, P5 and P7 in cells infected with white-tailed deer (CAD5_BV_WTD) prions (**c**–**e**), as well as with mule deer (CAD5_BV_MD) prions (**k**,**l**,**m**). (**a**,**i**) represent uninfected cell lysate at passage 3 (negative control). (**b**,**j**) 1% WTD- and MD -brain homogenate (BH) was used as positive control for the assay. No seeding activity was observed in non-reconstituted KO cells with either white-tailed deer (CAD5_KO _WTD) (**f**–**h**) or mule deer (CAD5_KO_MD) CWD prions (**n**,**o**,**p**).

The primary purpose behind introducing bank vole PrP was to use its promiscuous prion propagation to convert the designed platform into a more universal infection model. We tested for infection using cervid CWD prions since cervid prion infection models are relatively scarce. To demonstrate how the unrestrained nature of bank vole PrP can be used to develop a more ubiquitous infection platform, we tested two cervid prion isolates, white-tailed deer (WTD) and mule deer (MD) CWD prions, for prion infection in BVPrP expressing CAD5 (CAD5_BV) and MEF (MEF_BV) cells. As previously, cells were infected with 1% brain homogenate from terminal ill mice infected with WTD or MD CWD prions. However, since these cells represent an uncloned population which is not enriched for highly susceptible cells, we did not observe a PrP^Sc^ signal in Western blot analysis, prompting us to look for a more sensitive assay for detection of infection. The cells were therefore tested for prion seeding and conversion activity, using the real-time quaking induced conversion (RT-QuIC) assay. This assay measures amplification of PrP^Sc^ by monitoring real time amyloid formation as measured by increasing thioflavin fluorescence^44^. In order to further increase the sensitivity of detection, PrP^Sc^ in postnuclear cell lysates from infected and control cells was precipitated by sodium phosphotungstic acid (NaPTA). The use of NaPTA precipitated seed and recombinant mouse PrP as a substrate has been shown to be very effective in detection of CWD prions^45–47^. Uninfected cells were used as negative control (Fig. 5a,i) and for calculating the threshold cut-off values, while 1% BH after NaPTA precipitation was used as positive control for the assay (Fig. 5b,j). Positive seeding activity was detected from passage P3 to P7 in both CAD5_BV and MEF_ BV cells infected with WTD or MD CWD prions. CAD5_BV cells showed a strong RT-QuIC signature at least up to a dilution of 10^−3^ with WTD CWD (Fig. 5c–e) as well as for MD prions (Fig. 5k–m). The absence of seeding activity in all three dilutions in non-reconstituted CAD5 KO cells infected with WTD (Fig. 5f–h) or MD prions (Fig. 5n–p) confirmed that there was no residual inoculum or background noise in these cells, and clearly KO cells had a resistant phenotype. The positive seeding activity in CAD5_BV cells with both CWD prion isolates was tested and confirmed up to passage 9 (Fig. S4).

**Figure 6:**
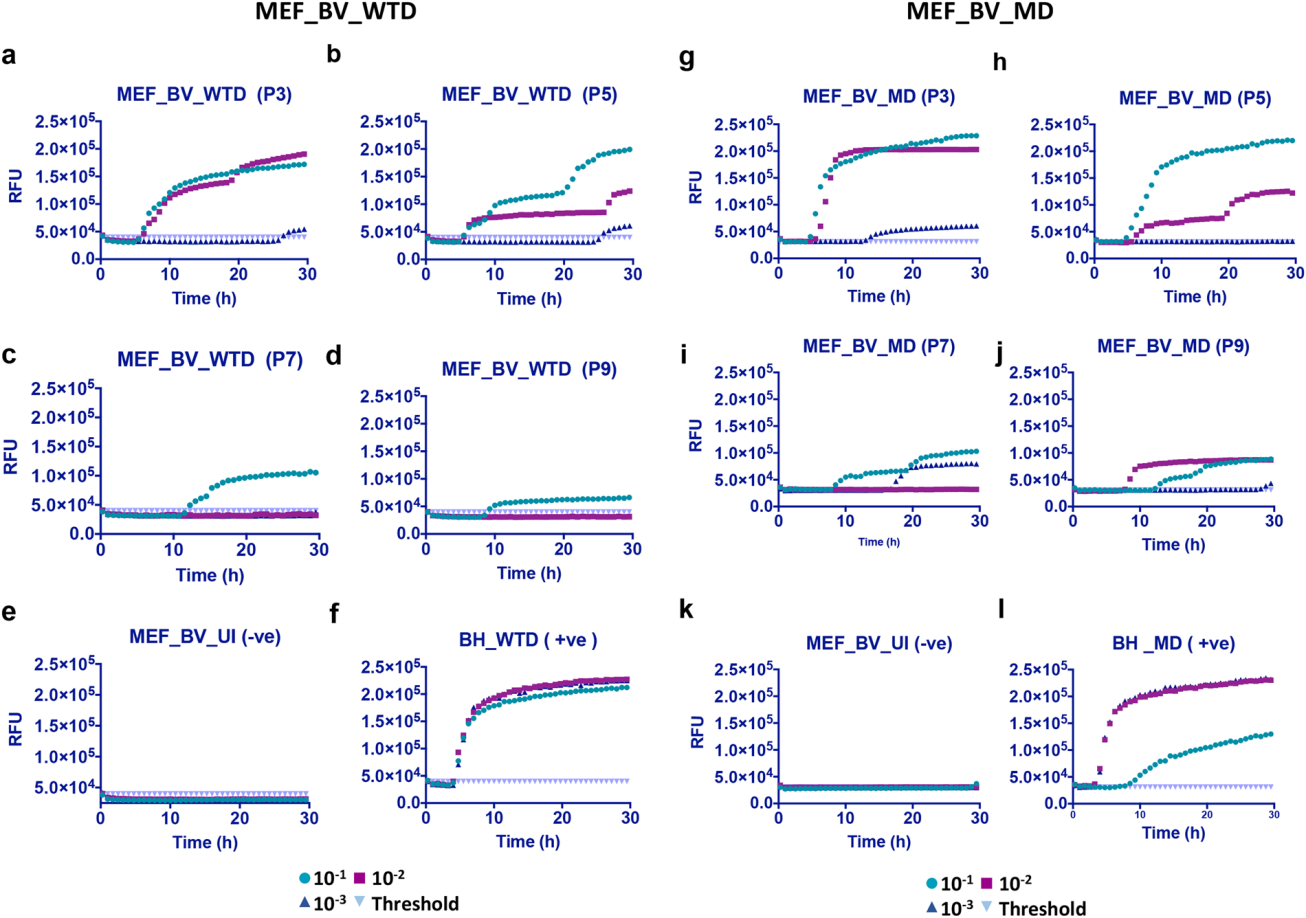
RT-QuIC analysis showing positive prion seeding activity in MEF_BV cells infected with white-tailed deer and mule deer CWD prions. Cell lysates were collected and processed for RT-QuIC analysis as done for Fig. 5. A positive seeding activity was seen at passage P3 to P9 in cells infected with white-tailed deer (MEF_BV_WTD) prions (**a**–**d**) as well as with mule deer (MEF_BV_MD) CWD prions (**g**–**j**). (**e**,**k**) represent uninfected cell lysate at passage 3 (negative control). (**f**,**l**) Represent 1% WTD- and MD-brain homogenate (BH) used as positive control for the assay.

MEF_BV cells were found to be positive for 10^−1^ or 10^−2^ dilutions with both WTD (Fig. 6a–d) and MD prions (Fig. 6g–j). As previously the negative controls (Fig. 6e,k) were used for calculating the cut-off values while 1% BH was used as positive control for the assay (Fig. 6f,l). However, the seeding activity was lower than that observed in CAD5_BV cells.

**Figure 7:**
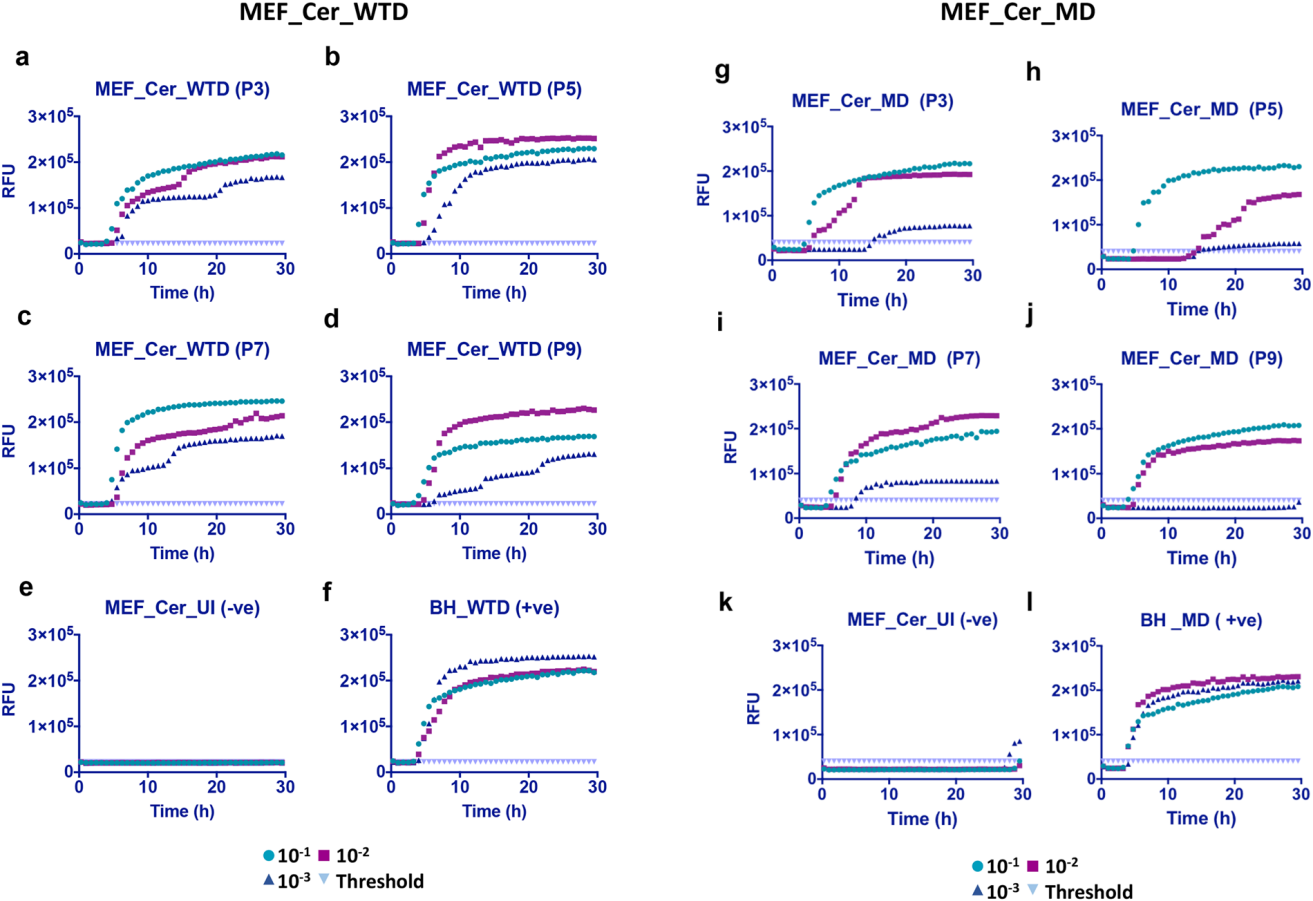
RT-QuIC analysis showing positive prion seeding activity in MEF_Cer cells infected with white-tailed deer and mule deer CWD prions. Cell lysates were collected at different passages (P3 to P9) post-infection and RT-QuIC assay was setup as done earlier. (**a**–**d**) Show a positive seeding activity from passage P3 to P9 in cells infected with white-tailed deer (MEF_Cer_WTD) CWD prions as well as with mule deer (MEF_Cer_MD) prions (**g**–**j**). (**e**,**k**) Represent uninfected cell lysate at passage 3 (negative control). (**f**,**l**) Represent 1% WTD- and MD -brain homogenate (BH) used as positive control for the assay.

So far we had two reconstituted cell lines expressing bank vole PrP which showed infection with two CWD isolates. To analyze the relative efficacy of the bank vole PrP template we decided to express cervid PrP in MEF cells and look for seeding activity with the two CWD prion isolates. MEF_KO cells were reconstituted with WTD cervid-PrP using lentiviral transduction as described earlier. These MEF_Cer cells were infected with WTD and MD prions and postnuclear lysates were collected and processed for RT-QuIC assay. As compared to the MEF_BV expressing cells, these cells gave a much stronger seeding activity in all three tested dilutions (10^−1^–10^−3^) with both prion isolates (Fig. 7a–d,g–j). The negative controls (Fig. 7e,k) were used for calculating the cut-off values while 1% BH was used as positive control for the assay (Fig. 7f,l). The fact that a sequence-matched PrP substrate/PrP^Sc^ template combination helped in recovering the infection efficiency shows that the host cell background had no role in the poorer seeding activity observed earlier. As previously, the MEF KO cells did not show any seeding activity when inoculated with WTD CWD prions, confirming the resistant phenotype and excluding that signals observed in reconstituted cells originate from inoculum (Fig. S5). Clearly MEF cells remain suitable candidates for the design of efficient infection platforms.

In summary, our data show that the engineered cell lines can stably express bank vole and cervid PrPs and have the ability to propagate CWD prions upon challenge when tested in RT-QuIC.

## Discussion

The limited availability of versatile cell culture platforms is a major bottleneck in comparative studies focusing on the role of prion protein polymorphisms and species-specific variations. This is especially true for new and newly emerging prion diseases like CWD where the scarcity of robust cellular models prevents *in vitro* studies on the role of CWD strain specific barriers and cervid PrP polymorphisms in disease propagation. Transformed primary culture brain cells from a mule deer were the first cell line reported as persistently infected with CWD prions^48^, but only after dilution cloning for persistently infected clones (with 1/51 positive). Later analysis showed that these cells are most likely brain tissue-derived fibroblasts. More recently, differentiated neurospheres from elk PrP-overexpressing mice were shown to be susceptible to infection with CWD prions^49^. Although not involving single cell cloning, this is a fairly complex strategy that requires growing primary cultures from transgenic mice and hence limits its applicability. Interestingly, a rabbit kidney cell line (RK13) stably transfected with cervid PrP^c^ has been shown to propagate CWD prions and harbor persistent infection^22,50^. RK13 cells do not express rabbit PrP as detectable at the mRNA and protein level, can be reconstituted with exogenous PrP without the need of removing endogenous PrP expression, and showed persistent prion infection for various types of prions upon inoculation with brain homogenates^22^. Still this usually needs a single cell cloning approach for persistently infected cells, and in case of elk-PrP expressing cells needed stable co-transfection with a plasmid expressing HIV GAG precursor protein^22,50^.

Attempts to extend this approach to murine cells like N2a expressing elk-PrP were unsuccessful^22^. We postulate that the presence of expression of endogenous mouse PrP, rather than host cell specific background or co-factors, is responsible for this non-permissiveness to cervid prions. To test this hypothesis we created a PrP null background (PrP^−/−^) in murine cells, so that the endogenous mouse PrP expression would not compromise the transgenic PrP substrate and negatively impact its prion infection efficiency by competition or trans-dominant negative inhibition^51^. The choice of CAD5 and MEF cells was dictated by their excellent susceptibility to prion infection and robustness of infection^31,52,53^. Ensuring complete knock out at the genomic locus turned out to be a more complex problem in cells like CAD5 having multiple alleles as compared to MEF cells. We observed that the obtained CAD5 single cell clones carried different mutations in their exon 3 locus indicating aneuploidy/multiploidy of the *Prnp* gene and all these modifications acted in concert to block the expression of the endogenous native PrP. In line with this, we confirmed the presence of multiple chromosomes in CAD5 cells by chromosome analysis. Thus, the use of paired gRNAs was extremely efficacious in generating a combination of disruptions at the gRNA target sites leading to a PrP null background. The successful infection of these cells by CWD prions, upon stable reconstitution by bank vole and/or cervid PrP, validated our hypothesis. This opens up the possibility of genome editing of a large number of susceptible cell lines which were earlier considered to be non-permissive to infection by multiple prion strains, and developing them into transgenic infection platforms.

The major strength of the outlined strategy is its versatility and the significant potential of extending it to construct cell lines which can handle a broader range of prion infections. Successful *Prnp* genome editing can be done on both neuronal and non-neuronal cell lines, as shown here by us, in order to determine the most suitable host for prion infection. While the role of host cell physiology in susceptibility and sustainability of infection is unclear, it is well established that certain cell lines are more permissible to infection with specific groups of prions. We may thus need to explore a panel of hosts with varying genetic backgrounds to develop a comprehensive cellular toolkit for molecular understanding of both existing and newly emerged prion diseases. The choice of the PrP^c^ substrate is also critical. Even though “promiscuous” PrPs like bank vole PrP are suitable for a range of prions, they may not necessarily provide optimum infectivity as was observed here with MEF cells. Very recently, the successful engineering of CAD5 cells for the stable propagation of hamster prions was shown^54^.

We used stable lentiviral transduction for heterologous gene delivery, but it would be equally possible to extend the CC9 knock out approach to a precise gene replacement strategy where the foreign prion protein gene can be inserted at the exact locus of its native counterpart. This would ensure that the expression of this gene would take place in its natural genomic landscape under the endogenous *Prnp* promoter, with comparable levels of expression when this is needed for quantitative assessments. Another useful extension of this strategy which employs the same work flow is in the study of CWD polymorphisms and their impact on CWD prion infectivity. Targeted single base pair changes can be carried out *in vitro* using CC9 rather than re-constructing the cellular platform from scratch^55,56^. This would ensure uniformity of the infection platform and help to insulate the effect of polymorphic alleles from potential variations due to host background or expression differentials.

Of note, the use of RT-QuIC allows highly sensitive detection of prion conversion and prion propagation in the uncloned population of inoculated cells, and discriminates within 3 passages between new propagation and incoming prion inoculum. Detection of newly converted PrP^Sc^ is usually done by immunoblot analysis and requires a high level prion infection and propagation which very often takes several passages post inoculation. Although we were unable to detect a signal in standard immunoblot analysis, the cells showed a clear and sustained infection in RT-QuIC for at least nine passages. This provides a reliable proof-of-concept that at least a certain portion of inoculated cells propagated prions, and future single cell cloning can be used for enrichment of highly susceptible and persistently prion propagating cells. Such single cell clones would have higher infection levels, be positive in immunoblot for PrP^Sc^, and not show the gradual decrease in seeding activity that was observed with passaging in this study. Clones also can be cured with anti-prion compounds and tested for re-infection potential. If positive, these highly susceptible cells can be used as acceptor cells in scrapie-cell assay which allows quantitative assessment of infection traits^30^.

Taken together, this study helps to establish a work flow for constructing cell culture models for transgenic prion infections. The versatility of such platforms opens up the possibility of studying a wider range of prion strains and the impact of *Prnp* polymorphisms on prion strain barriers.

## Materials and Methods

### Cloning sgRNA into CRISPR plasmids for co-expression with Cas9

‘CRISPR Design Tool’ (http://crispr.mit.edu) was used to scan the murine *Prnp* exon 3 sequence and gRNA1 (position 88–108) and gRNA2 (position 269–289) were selected (Supplementary Table 1). The gRNA oligonucleotide pairs were synthesized from the core DNA services at the University of Calgary and were customized to include overhangs compatible for ligation into CRISPR plasmids linearized by digestion with *Bbs*I. The pX458-GFP and pX459-puro plasmids were obtained from a non-profit plasmid share repository (Addgene, Cambridge, MA, USA). Oligonucleotides were phosphorylated with polynucleotide kinase and annealed to generate double stranded duplex molecules. gRNA1 and gRNA2 duplices were then inserted into the pX458 and pX459 plasmids, respectively, using T4 DNA ligase, and transformed into Stbl3 competent *E*. *coli* bacteria (Thermo Fisher Scientific). The correct insertion of gRNA into the CRISPR plasmids was confirmed by DNA sequencing.

### Maintenance of cell lines

CAD5 cells were a generous gift of Dr. S. Mahal (The Scripps Research Institute Florida)^29,30^. Cells were grown in Opti-MEM glutamax media (GIBCO, USA) containing 10% (*v/v*) bovine growth serum (Hyclone, USA). Immortalized mouse embryonic fibroblasts (MEF) were obtained from Dr. N. Mizushima (Tokyo Medical and Dental University, Tokyo, Japan) and grown in DMEM glutaMax media (GIBCO, USA) containing 10% fetal bovine serum (Sigma, USA)^31^. Human embryonic kidney (HEK-293FT) cells for lentiviral transduction were purchased from Invitrogen (Karlsruhe, Germany) and maintained in DMEM glutamax media (GIBCO, USA) containing 10% fetal bovine serum. All cells were grown at 37 °C in a 5% CO_2_ atmosphere.

### DNA delivery into cultured cells

Lipofectamine 2000 (Life Tech) was used to deliver gRNA plasmids (2.0 μg each) into CAD5 cells according to the manufacturer’s instructions and at a cell culture plate confluency of approximately 60–70%. 48 hours post transfection, cells were harvested and processed for GFP-FACS sorting. DNA delivery into MEFs by nucleofection was optimized using manufacturer’s instructions and the Lonza-MEF starter Kit-VPD-1006 and Amaxa 2b-Nucleofector device. MEF solution-2 and T-20 program were selected for optimal nucleofection. MEFs were grown in T25 flasks to a confluency of 60–70%. Two times 10^6^ cells per nucleofection were collected at 250 × g for 5 minutes and resuspended into pre-warmed S1-supplemented MEF solution-2. gRNA plasmids (each 2.0 µg) were added to the cells and incubated for 10 min on ice. The cells with DNA were transferred to a 100 µl nucleocuvette and electroporated using T-20 Nucleofector 2b program. Post pulse, 500 µl of pre-warmed growth media was added to the nucleocuvette and the cells were transferred to a 12 well plate. Forty-eight hours post nucleofection, cells were harvested and processed for GFP-FACS sorting.

### Isolation of clonal cell lines by FACS

Fluorescence-activated cell sorting (FACS) analysis was used to enrich the potential CRISPR-edited pool of cells. CAD5 and MEF cells were cultured and transfected as described above. Single cells were identified on the basis of FSC-A, FSC-W and SSC-A and GFP fluorescence measured with 488 nm excitation and emission detected at 530 nm. Cell sorting was performed on FACS Aria cell sorter (Becton Dickinson) at the University of Calgary flow cytometry core facility. Prior to sorting, the cells were detached from the plates using Trypsin-EDTA (Invitrogen), centrifuged, and resuspended in fresh FACS buffer (2.0% FCS in PBS). The cell suspensions were filtered through 35 μm mesh. Single cells were sorted onto individual wells of a 96 well plate containing Opti-MEM supplemented with 20% FCS or bovine growth serum (BGS), 100 units/ml penicillin and 100 mg/ml streptomycin. Single cell clones were allowed to proliferate and subsequently expanded in 6-well and 60 mm plates until cell numbers were sufficient for cell banking, genomic DNA extraction and immunoblot analyses. For MEF sorting, due to their larger size the cells were passed through a 130 µm nozzle and expanded to single cell clones using the above criteria.

### Detection of *indel* mutations by PCR

Genomic DNA was isolated from single cell clones using Quick Extract-DNA extraction solution according to the manufacturer’s instructions (Epicenter). The extracted DNA served as a template to amplify the *Prnp* gene with primers PrP-*F*/PrP-*R* (Supplementary Table 1) using Q5 DNA polymerase. The PCR conditions were 94 °C for 5 min, followed by 40 cycles of 94 °C for 45 s, 59 °C for 45 s, 72 °C for 60 s, and 7 min at 72 °C. The PCR products were purified using QIAquick PCR purification kit, cloned into pCR–blunt TOPO cloning kit. The ligated mixture was transformed into Stbl3 competent *E*. *coli* cells (Thermo Fisher Scientific). Plasmid DNA was isolated from twelve of the bacterial colonies per cell clone and sent for Sanger sequencing using the PrP-*F* and PrP-*R* primers.

### Construction of lentiviral expression vectors and transduction of KO cells with lentiviral particles

The wild-type mouse *Prnp* (MoPrP) coding sequence from pCDNA3.1-MoPrP plasmid was PCR amplified using LV-PrPF forward and LV-PrPR reverse primer (Supplementary Table 1). The 764 bp PrP amplicon was inserted at the *EcoR*I/*BamH*I sites of lentiviral vector pCDH-CMV-MCS-puro-TA-GFP (Clontech Laboratories, Inc., USA). pWP1 lentiviral plasmid (Addgene # 12254) was modified to insert *BamHI-SpeI-MluI-XmaI* (pWP1-mod-BMSX) cloning sites (HBI core facility, University of Calgary). Bank vole (BV) and cervid PrPs (WTD, 116G or 138N alleles) were PCR-amplified from pCDNA3.1-BVPrP/Cer-PrP and inserted at the *BamHI/MluI* sites of pWP1-mod. The lentiviral vectors containing the *Prnp* gene of interest (pWP1-BV, pWP1-WTD, pWP1-116G, pWP1-138N) were co-transfected using Lipofectamine LTX transfection reagent into HEK293FT cells along with Addgene packaging pPAX and envelop plasmids pMD2.G (www.addgene.org). 48 hours post transfection cell culture supernatants were collected and filtered (0.45 µm) to remove cellular debris and used for transduction of recipient cells. The supernatant containing the PrP-lentiviral particles was used to transduce CAD5-KO and MEF-KO cells. Five times 10^4^ cells were plated in a 12 -well plate. The next day cells were incubated with 4 μg/ml polybrene for 30 min before exposure to 30–50 µl of lentiviral particles (multiplicity of infection (M.O.I.) of 30–50) overnight. Twenty four hours later the media was replaced by fresh growth media. Stable Mo-PrP transductants were selected using puromycin selection. BV and cervid -PrP transductants were enriched using GFP sorting 48 hours post transduction. The sorted cells were passaged for several generations and a second round of FACS sorting was done to select for stable transductants. Western blotting was used to validate the expression of PrP in the stable cells.

### Primary prion infection of cells

The cells were infected with 1% brain homogenate of a terminally ill mouse infected with 22 L prions or CWD white–tailed deer (WTD) or mule deer (MD) prions. To prepare brain-homogenates, the brains from infected mice were homogenized in PBS at a final concentration of 10% (w/v) and stored at −80 °C. For primary prion infection, 1 × 10^6^ cells were seeded in a 6-cm culture dish. After 24 h, culture medium was removed and cells were incubated with 1% brain homogenate in appropriate serum-free culture medium (900 µl). After 5 hours, 1 ml complete culture medium was added to the cells. Twenty four hours later the cells were washed with 1X PBS and fresh culture medium was added. The cells were grown back to confluence and passaged further. For detection of PrP^Sc^ upon primary prion infection, cells were lysed at different passages. Lysates were used for PK-digestion, immunoblot analysis and RT-QuIC.

### Cell lysis, proteinase K (PK) digestion and immunoblot analysis

Cell lysis, PK profiling and immunoblot was performed as described previously^57^. Briefly, confluent cells were lysed in chilled cell lysis buffer (10 mM Tris-HCl, pH 7.5; 100 mM NaCl; 10 mM EDTA; 0.5% Triton X-100; 0.5% sodium deoxycholate (DOC)) for 10 minutes. For PK digestion, 50% of the cell lysate was incubated with 20 μg/ml PK (Roche, Germany) for 30 min at 37 °C. PK digestion was stopped with 0.5 mM pefabloc protease inhibitor (VWR), and the samples were precipitated with 5 volumes of methanol. For non-PK digested samples (−PK), pefabloc was added directly and samples precipitated with methanol. Precipitated proteins were re-suspended in appropriate volume of TNE buffer (50 mM Tris-HCl pH 7.5; 5 mM EDTA; 150 mM NaCl). Samples were run on 12.5% SDS-PAGE, and electro-blotted on Amersham Hybond P 0.45 PVDF membranes (Amersham, USA). The blots were developed using Luminata Western Chemiluminescent HRP Substrates (Millipore, USA). PrP^c^ and PrP^Sc^ was detected with anti-PrP monoclonal antibody (mAb) 4H11 as described previously^58^. Peroxidase-conjugated goat anti-mouse HRP secondary antibodies were from Jackson ImmunoResearch, USA. Anti-β-actin (Sigma Aldrich, USA) was used for loading control.

### Sodium phosphotungstic acid (NaPTA) precipitation for RT-QuIC assay

NaPTA precipitation of cell lysates and brain homogenates was done as described earlier^46^. Briefly, 100 μl of cell lysates from the infection experiments were mixed with N-lauryl-sarcosine at a final concentration of 2% and incubated at 37 °C and constant shaking at 1,400 rpm for 30 min. The samples were adjusted to 0.3% NaPTA by adding a stock solution containing 4% NaPTA and 170 mM MgCl_2_, pH7.4, incubated for 2 hours at 37 °C with constant shaking at 350 rpm. The samples were then centrifuged for 30 min at maximum speed (15,800 × g) at 4 °C. Pellets were washed using cell lysis buffer containing 0.1% N-lauryl-sarcosine, and resuspended in 1/10 of the original sample volume in RT-QuIC seed dilution buffer. 1% BH and lysates from non-infected (or KO) cells precipitated with NaPTA were used as positive and negative controls, respectively, in RT-QuIC.

### Real-time quaking inducing conversion assay (RT-QuIC)

Recombinant mouse prion protein was prepared as described earlier^52,59^. The real-time quaking induced conversion assay was performed as described earlier^60^. Briefly, reactions were set up in assay buffer containing 20 mM Na-phosphate, pH 7.4, 300 mM NaCl, 1 mM EDTA, 10 μM Thioflavin T and 0.1 mg/ml of mouse- rPrP substrate. Ninety-eight μl aliquots were added to the wells of a black-walled 96-well optical bottom plate (Nalge Nunc International, Nunc). Tenfold serial dilutions of NaPTA precipitated cell lysates or brain homogenate were prepared in seed buffer containing 0.02% SDS. Quadruplicate reactions were seeded with 2 µl of each dilution for a final reaction volume of 100 µl. Plates were sealed with Nunc Amplification Tape (Nalge Nunc International) and incubated in a FLUOstar Omega plate reader for 30 hours at 42 °C, with cycles of 60 s shaking and 60 s rest throughout the incubation. ThT fluorescence was measured (450 nm excitation and 480 nm emission) every 15 min. RT-QuIC data were averaged from four replicate wells and average values plotted against reaction time. Samples were scored positive if at least 50% of replicates reached a ThT fluorescence cut-off, which was calculated based on the average ThT fluorescence plus 5 x standard deviation.

### Ethics statement

All animal experiments used for generating infectious material (brain homogenates) were performed strictly following the Canadian Council for Animal Care (CCAC) guidelines and were approved by the University of Calgary Health Sciences Animal Care Committee (HSACC). The experiments involving the propagation of 22 L prions and cervid prion strains in C57Bl/6 mice (obtained from Charles River Laboratories, USA) were approved under protocol number AC18-0047.

## Supplementary information

Supplementary data
