# Supplementary material for: Gene-edited murine cell lines for propagation of chronic wasting disease prions
Source: Sci Rep. 2019 Aug 1;9:11151. doi: 10.1038/s41598-019-47629-z (PMC6673760; doi:10.1038/s41598-019-47629-z)

Gene-edited murine cell lines for propagation of chronic wasting disease prions

Rupali Walia, Cheng Ching Ho, Chi Lee, Sabine Gilch and Hermann M. Schatzl

Supplementary Data

```
CAD5_WT         CCTGGAGGGTGGAACACCGGTGGAAGCCGGTATCCCGGGCAGGGAAGCCCTGGAGGCAAC
CAD5_KO2A       CCTGGAGGGTGGAACACCGGTG--------------------------------------
CAD5_KO2B       CCTGGAGGGTGGAACACCGGTAT----CGGTATCCCGGGCAGGGAAGCCCTGGAGGCAAC
CAD5_KO2C       CCTGGAGGGTGGAACACCGGTAT----------CCCGGGCAGGGAAGCCCTGGAGGCAAC
                *********************.

CAD5_WT         CGTTACCCACCTCAGGGTGGCACCTGGGGGCAGCCCCACGGTGGTGGCTGGGGACAACCC
CAD5_KO2A       ------------------------------------------------------------
CAD5_KO2B       CGTTACCCACCTCAGGGTGGCACCTGGGGGCAGCCCCACGGTGGTGGCTGGGGACAACCC
CAD5_KO2C       CGTTACCCACCTCAGGGTGGCACCTGGGGGCAGCCCCACGGTGGTGGCTGGGGACAACCC

CAD5_WT         CATGGGGGCAGCTGGGGACAACCTCATGGTGGTAGTTGGGGTCAGCCCCATGGCGGTGGA
CAD5_KO2A       ------------------------------------------------------------
CAD5_KO2B       CATGGGGGCAGCTGGGGACAACCTCATGGTGGTAGTTG----------------------
CAD5_KO2C       CATGGGGGCAGCTGGGGACAACCTCATGGTGGTAGTTG----------------------

CAD5_WT         TGGGGCCAAGGAGGGGGTACCCATAATCAGTGGAACAAGCCCAGCAAACCA
CAD5_KO2A       ---------GAAGGGGGTACCCATAATCAGTGGAACAAGCCCAGCAAACCA
CAD5_KO2B       ------------GGGTCAGCCCCATGGCGGTGGAACAAGCCCAGCAAACCA
CAD5_KO2C       ------------GGGTCAGCCCCATGGCGGTGGAACAAGCCCAGCAAACCA
                            ***  :.***.::. *.**********************
```

Supplementary Figure S1a: *Indel* signatures in CAD5-KO2 clone.

Genomic PCR was done from CAD5-KO2 cell clone using PrP-*F* and PrP-*R* primers and cloned into a bacterial mini library . Sequence comparison of ten bacterial inserts with the wild type control (CAD5_WT) shows : CAD5_KO2A having a large deletion between gRNA1 and gRNA2 (the same sequence appeared in 7/10 bacterial clones) while CAD5_KO2B and CAD5_KO2C had disruptions/smaller deletions at the gRNA1and gRNA 2 site and appeared in 2/10 and 1/10 sequences respectively. Yellow and blue regions represent the gRNA1 and gRNA2 sites, respectively. Sequence in red represents *indels*.

```
CLUSTAL 2.1 multiple sequence alignment


MEF_KO1         CCTGGAGGGTGGAACACCGGTGGAAGCCGGTATCCCGGGCAGGGAAGCCCTGGAGGCAAC
MEF_KO2         CCTGGAGGGTGGAACACCGGTGGAAGCCGGTATCCCGGGCAGGGAAGCCCTGGAGGCAAC
MEF_WT          CCTGGAGGGTGGAACACCGGTGGAAGCCGGTATCCCGGGCAGGGAAGCCCTGGAGGCAAC
                ************************************************************

MEF_KO1         CGTTACCCACCTCAGGGTGGCACCTGGGGGCAGCCCCACGGTGGTGGCTGGGGACAACCC
MEF_KO2         CGTTACCCACCTCAGGGTGGCACCTGGGGGCAGCCCCACGGTGGTGGCTGGGGACAACCC
MEF_WT          CGTTACCCACCTCAGGGTGGCACCTGGGGGCAGCCCCACGGTGGTGGCTGGGGACAACCC
                ************************************************************

MEF_KO1         CATGGGGGCAGCTGGGGACAACCTCATGGTGGTAGTTGGGGTCAGCCCCATGGCGGTGGA
MEF_KO2         CATGGGGGCAGCTGGGGACAACCTCATGGTGGTAGTTGGGGTCAGCCCCATGGCGGTGGA
MEF_WT          CATGGGGGCAGCTGGGGACAACCTCATGGTGGTAGTTGGGGTCAGCCCCATGGCGGTGGA
                ************************************************************

MEF_KO1         TGGGGCCAAGGAAGGGGGTACCCATAATCAGTGGAACAAGCCCAGCAAACCAAAAACCAA
MEF_KO2         TGGGGCCAAG-----GGGTACCCATAATCAGTGGAACAAGCCCAGCAAACCAAAAACCAA
MEF_WT          TGGGGCCAAGG-AGGGGGTACCCATAATCAGTGGAACAAGCCCAGCAAACCAAAAACCAA
                **********     *********************************************
```

Supplementary Figure S1b: ***Indel*** **signature in MEF-KO clones.**

Genomic PCR was done from a representative CC9 clonal isolate of MEF cells using PrP-*F* and PrP-*R* primers. Sequence comparison of PCR amplicons (cloned into a bacterial mini library - KO1 and KO2) with the wild type (MEF_WT) shows *indels* at the gRNA2 target site. Yellow and blue regions represent the gRNA1 and gRNA2 sites, respectively. Sequence in red represents *indels*.

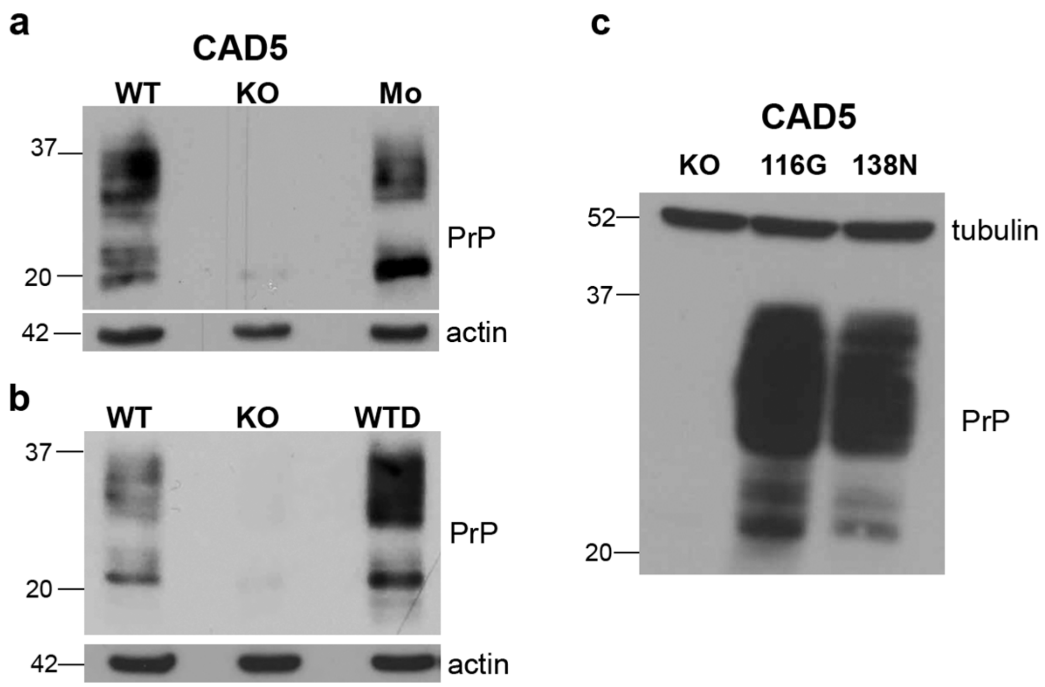

Supplementary Figure S2: Stable reconstitution of CAD5 PrP$^{-/-}$ cells (KO2) with MoPrP and cervid WTD, 116G, and 138N PrP using lentiviral transduction.

(a) Western blot analysis showing expression of Mo-PrP in transduced cells. **(b)** Western blot showing the expression of wild-type WTD-PrP (white-tailed deer) in transduced cells. **(c)** Western blot showing expression of 116G and 138N-PrP in transduced cells. WT represents the parental wild-type CAD5 cells expressing murine PrP and KO represents the CAD5 knock-out background (KO2). The blots were probed with anti-PrP mAb 4H11 and actin or tubulin was used as a loading control

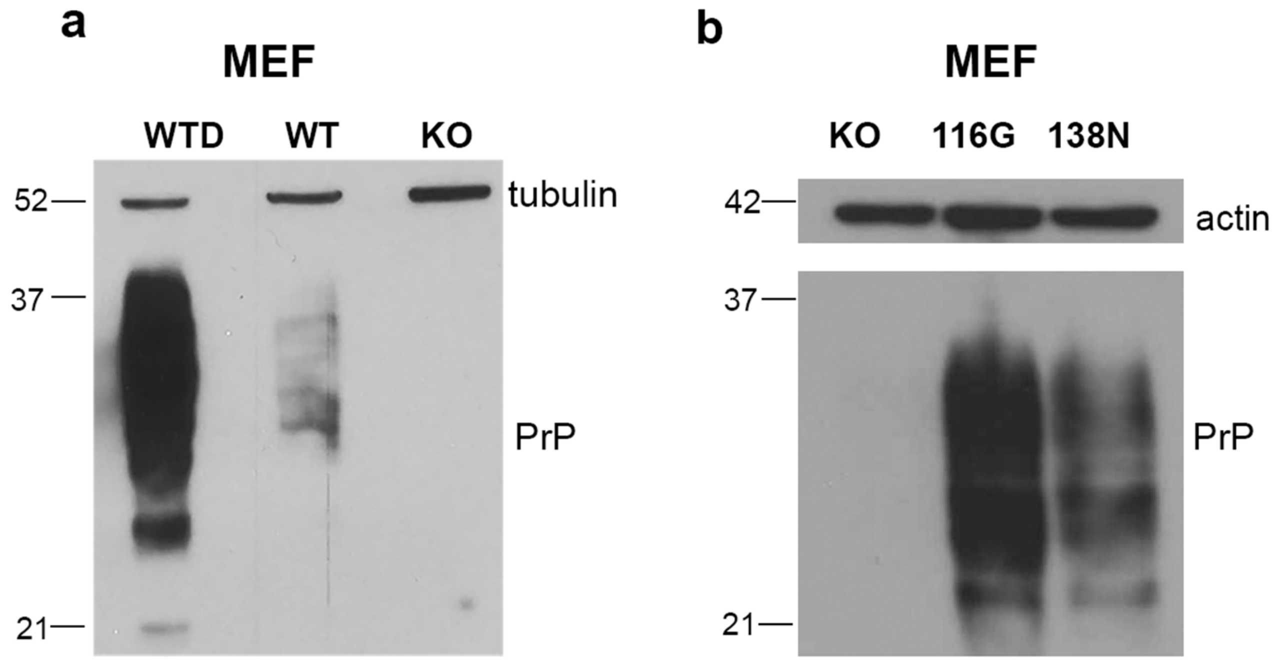

Supplementary Figure S3: Stable reconstitution of MEF PrP$^{-/-}$ cells (KO1) with cervid WTD, 116G and 138N polymorphic PrP variants using lentiviral transduction.

(a) Western blot analysis showing the expression of wild type WTD-PrP (white–tailed deer) in transduced cells. **(b)** Western blot showing expression of 116G and 138N-PrP in transduced cells. WT represents the parental MEF wild-type cells expressing murine PrP and KO represents the MEF knock out cells (KO1). The blots were probed with anti-PrP mAb 4H11 and tubulin was used as a loading control.

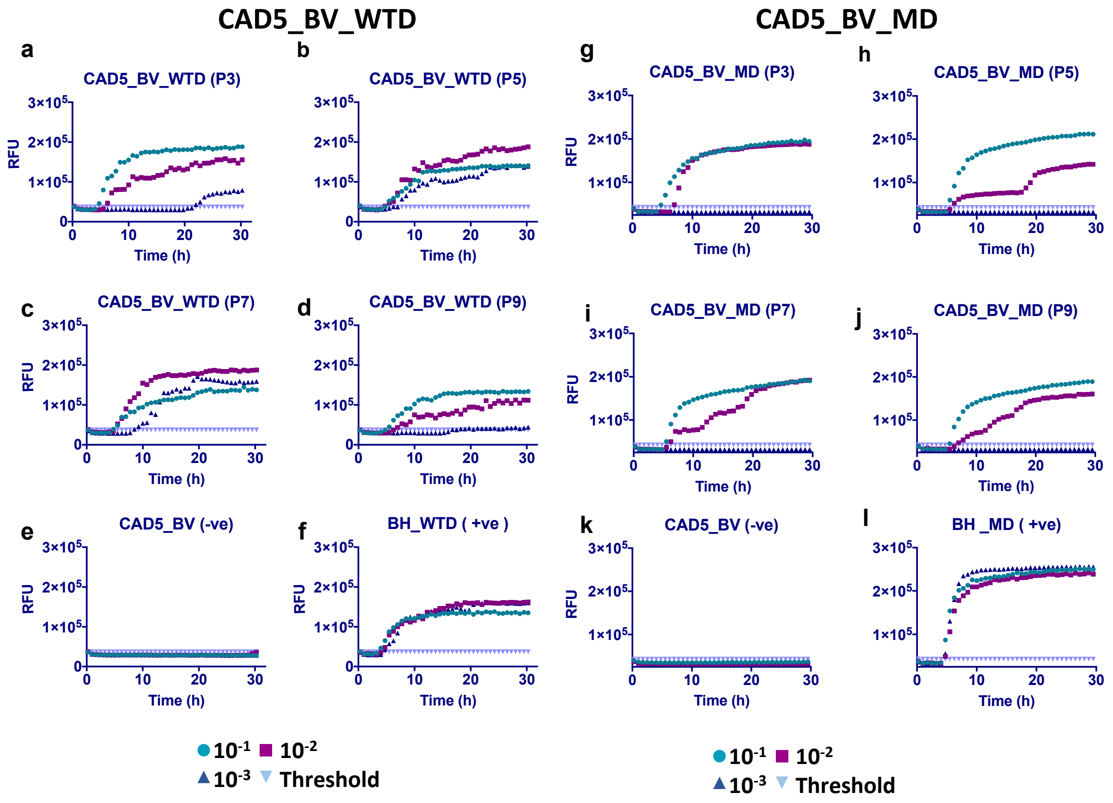

Supplementary Figure S4: RT-QuIC analysis showing positive prion seeding activity in CAD5_BV cells with white–tailed deer (WTD) and mule deer (MD) CWD prions.

RT-QuIC assay was setup as done in Fig. 5. A positive seeding activity was seen up to passage P9 in cells infected with white-tailed deer (CAD5_BV_WTD) prions **(a-d)** as well as with mule deer (CAD5_BV_MD) CWD prions **(g-j)**. **(e, k)** represent uninfected cell lysate at passage 3 (negative control). **(f, l)** 1% WTD- and MD-brain homogenate (BH) was used as positive control for the assay.

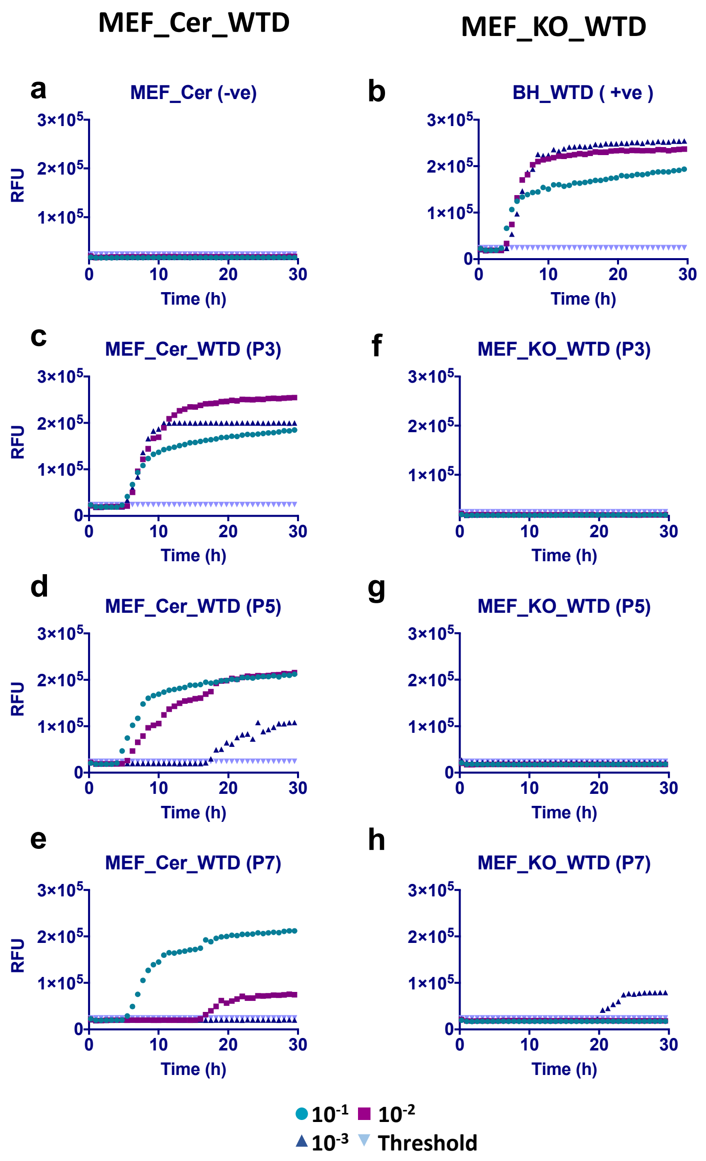

Supplementary Figure S5: RT-QuIC analysis showing positive prion seeding activity in MEF_Cer cells infected with WTD CWD prions and absence of seeding activity in infected knock out cells (MEF_KO_WTD).

RT-QuIC assay was setup as done in Fig. 5. A positive seeding activity was seen in passage P3-P7 in cells infected with white-tailed deer (MEF_Cer_WTD) prions **(c-e) .** No seeding activity was observed in infected non-reconstituted knock out (MEF_KO_WTD) cells **(f-h*)**. **(a)** represents uninfected cell lysate at passage 3 (negative control). **(b)** 1% WTD-brain homogenate (BH) was used as positive control for the assay.

* Sample h is negative as only one out of four replicates showed a weak signal . Samples are scored positive when at least 50% of the replicates (≥2 out of 4) reached a ThT fluorescence cut-off.

Supplementary Table S1: Oligonucleotides and primers used in this study

No.	Oligo/ Primer	Sequence
1	gRNA1	GGTGGAACACCGCTGGAAGC
2	gRNA2	GATTATGGGTACCCCCTCCT
3	PrP*F*	ATGGCGAACCTTGGCTACTGGCTG
4	PrP*R*	TCATCCCACGATCAGGAAGATG
5	LV- Mo-PrP*F*	TTTTTGAATTCGCCACCATGGCGAACCTTGGCTACTGGCTG
6	LV- Mo-PrP*R*	TTTTGGATCCTCATCCCACGATCAGGAAGATG
7	LV- BV-PrP*F*	GATGATGGATCCATGGCGAACCTCAGCTACTG
8	LV- BV-PrP*R*	ACGCGTTCATCCCACGATCAGGAAGA
9	LV- Cer-PrP*F*	GATGATGGATCCATGGTGAAAAGCCACATAGGC
10	LV- Cer-PrP*R*	ACGCGTCTATCCTACTATGAGAAAAAT

Gene-edited murine cells for propagation of chronic wasting disease prions.

Rupali Walia, Cheng Ching Ho, Chi Lee, Sabine Gilch and Hermann M. Schatzl

Supplementary: Original Blots

Figure 2d, 2e, 2f

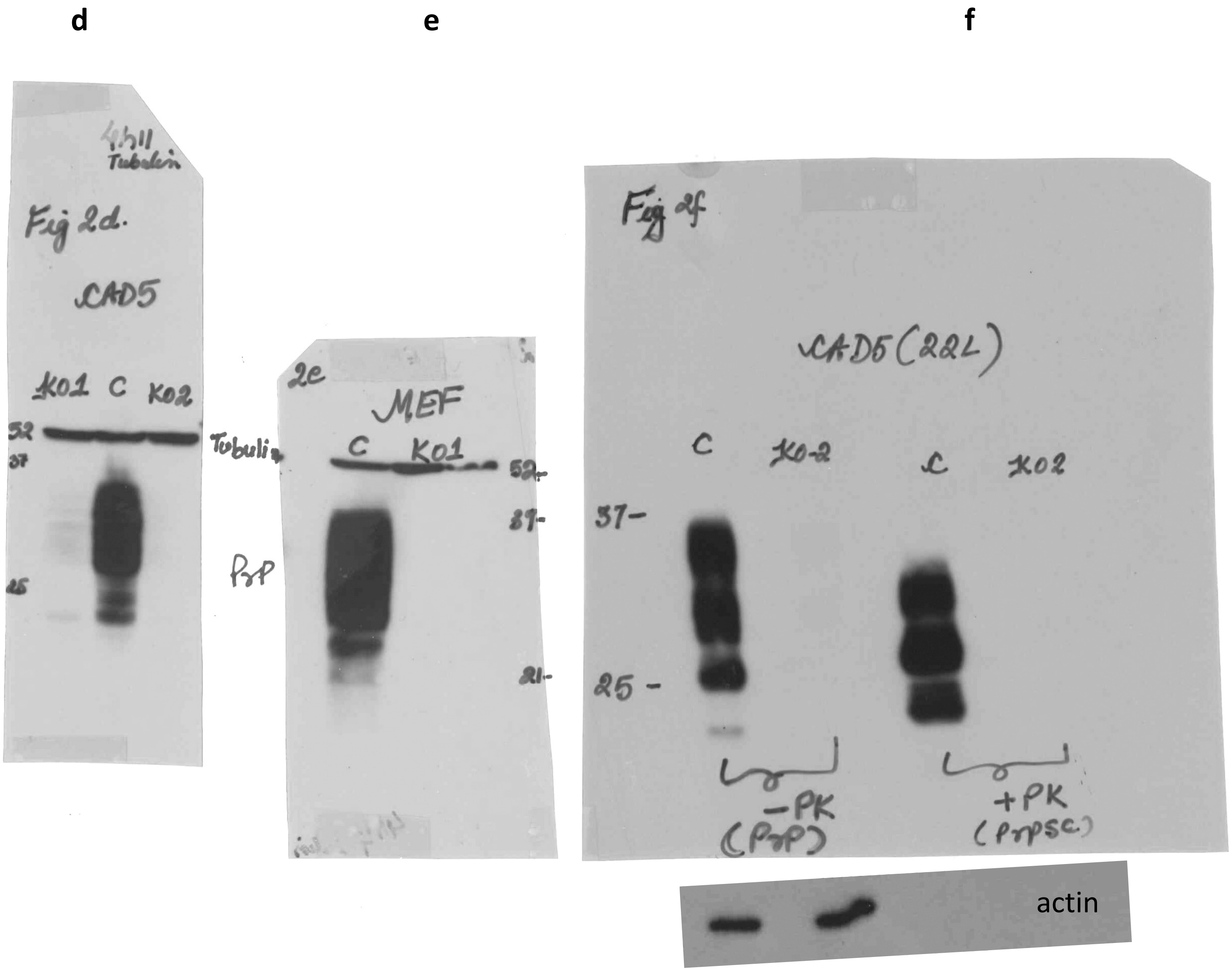

Figure 3c , 3f

c

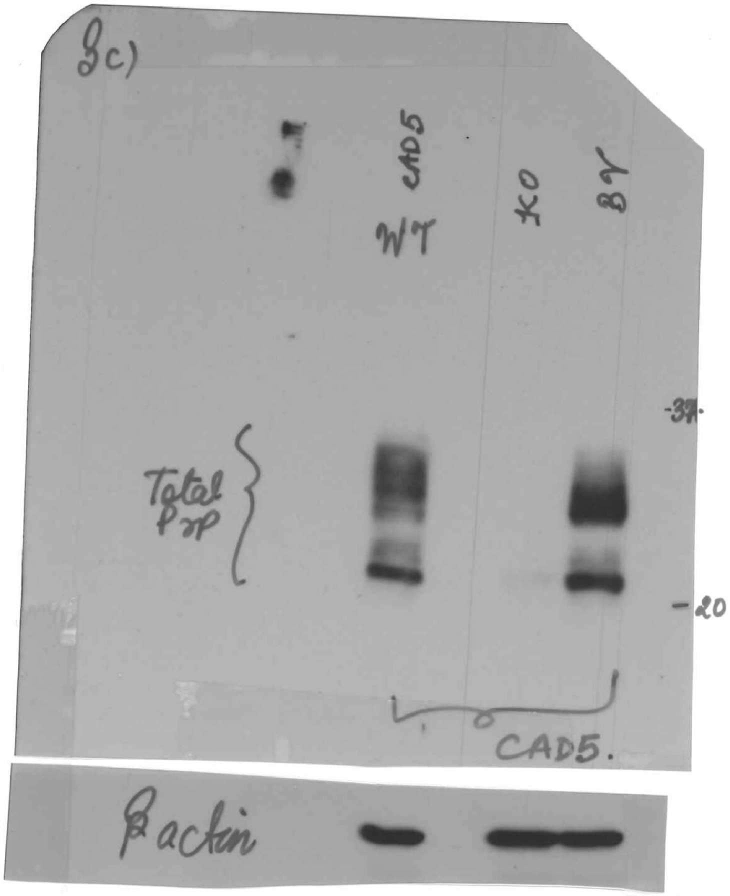

f

Multiple exposure for Fig 3f

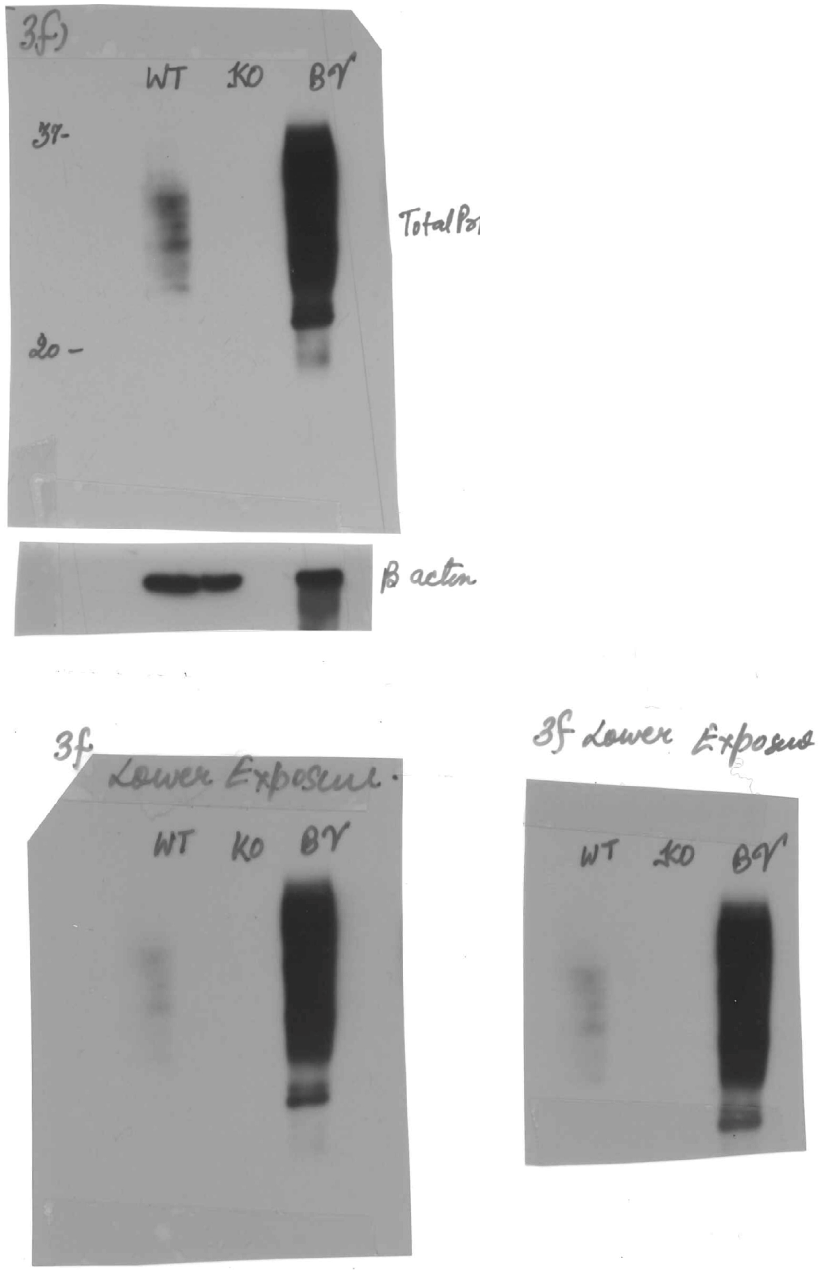

Figure 4a , 4b

Multiple exposure for Fig 4a and 4b

a

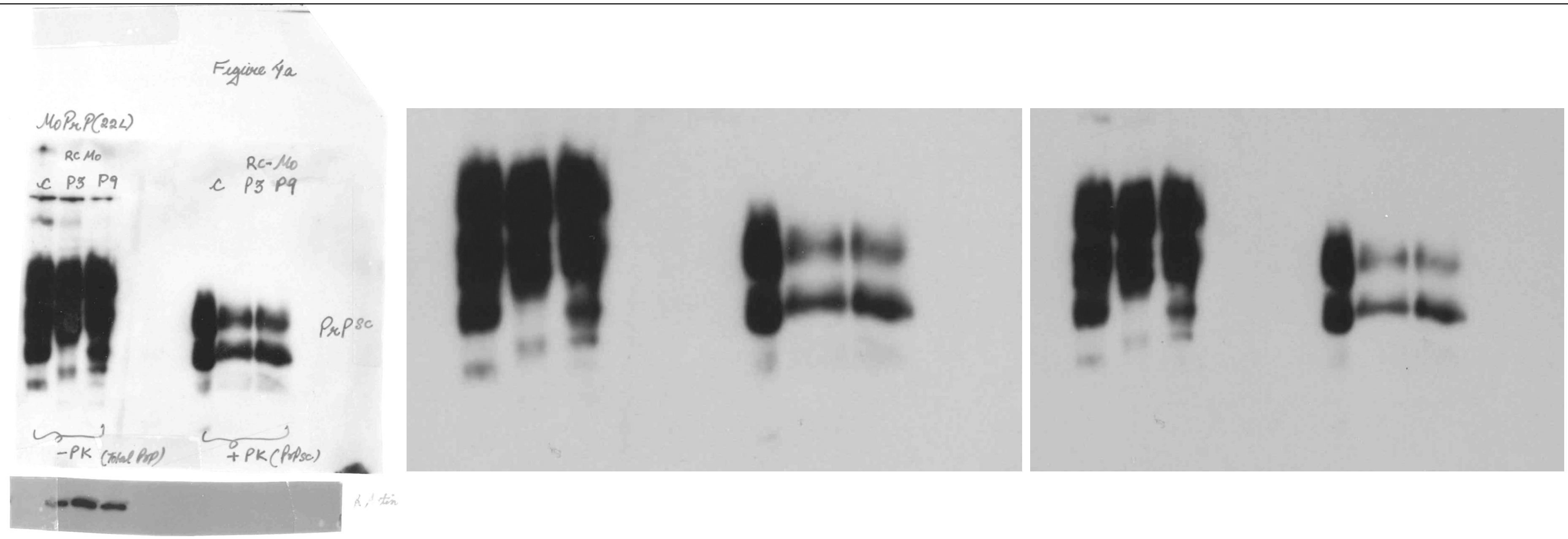

b

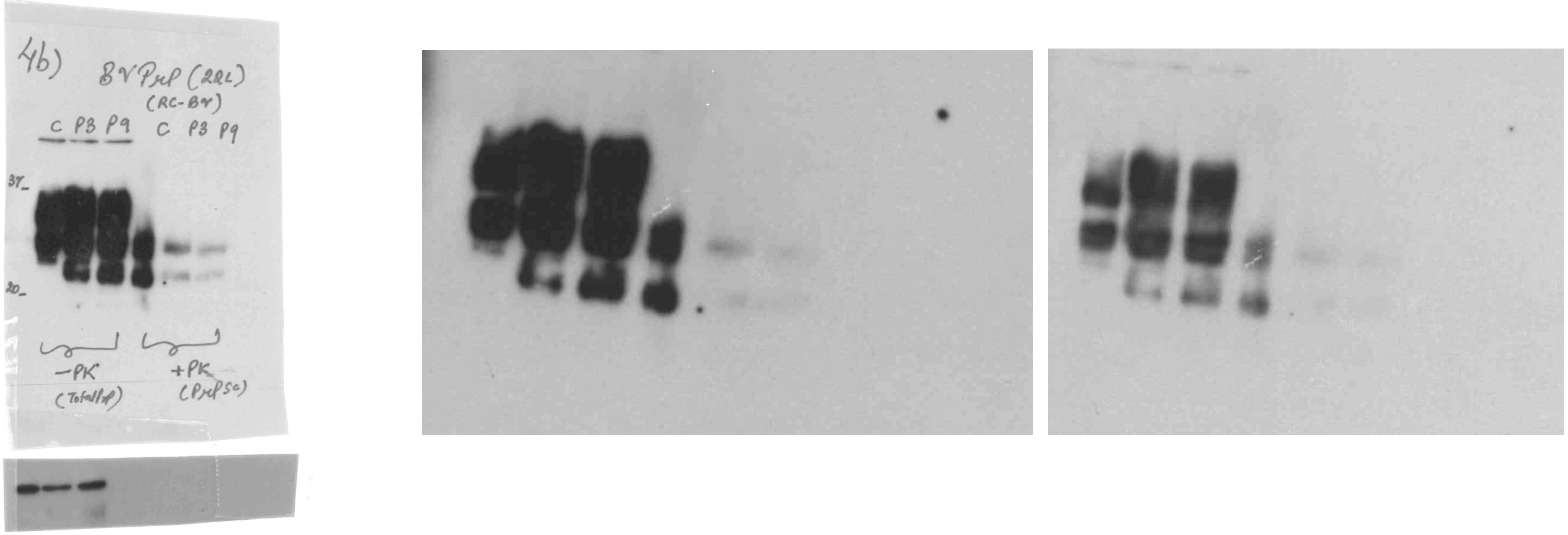

Figure S2 a, b, c

a

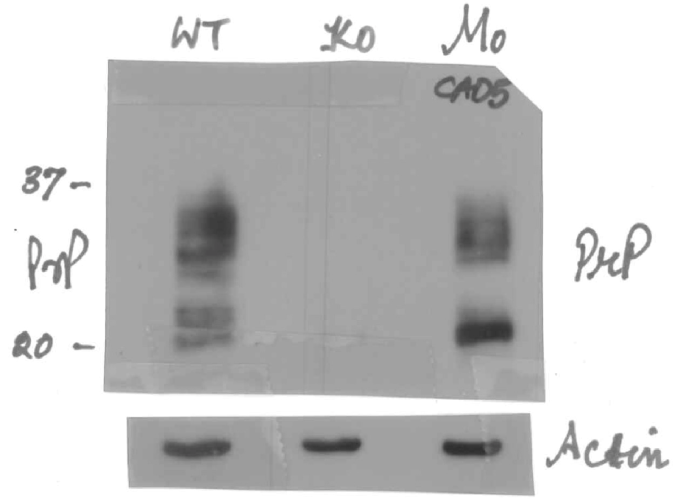

b

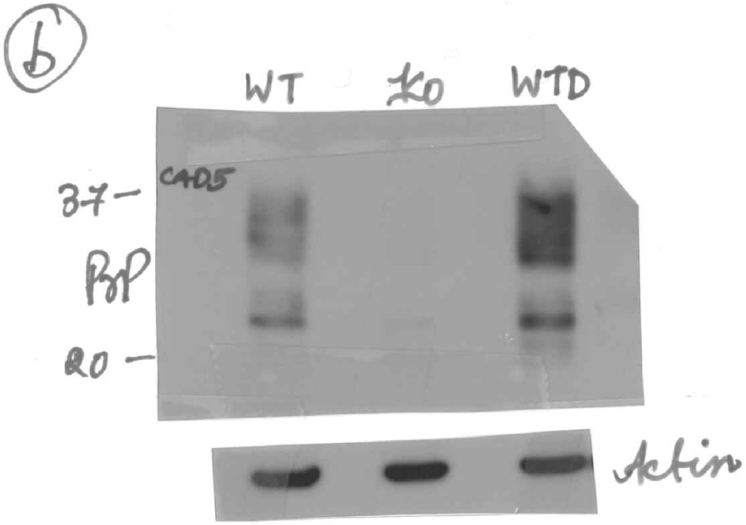

c

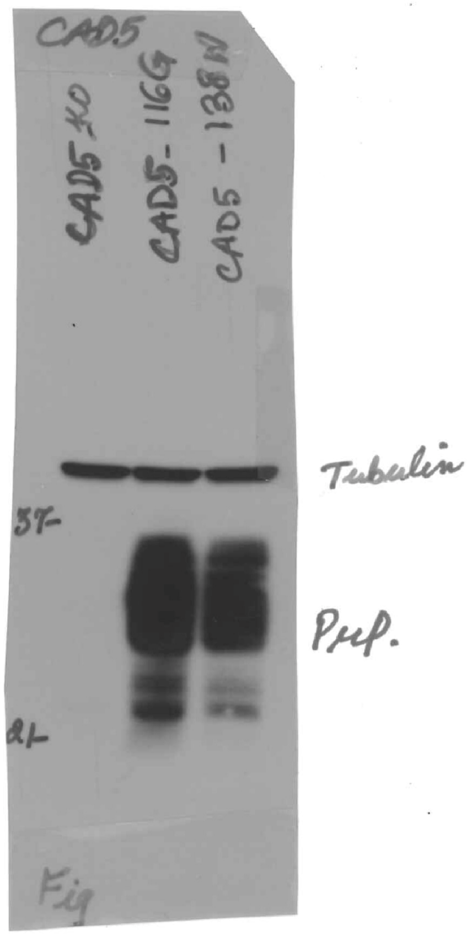

Figure S3 a, b

a

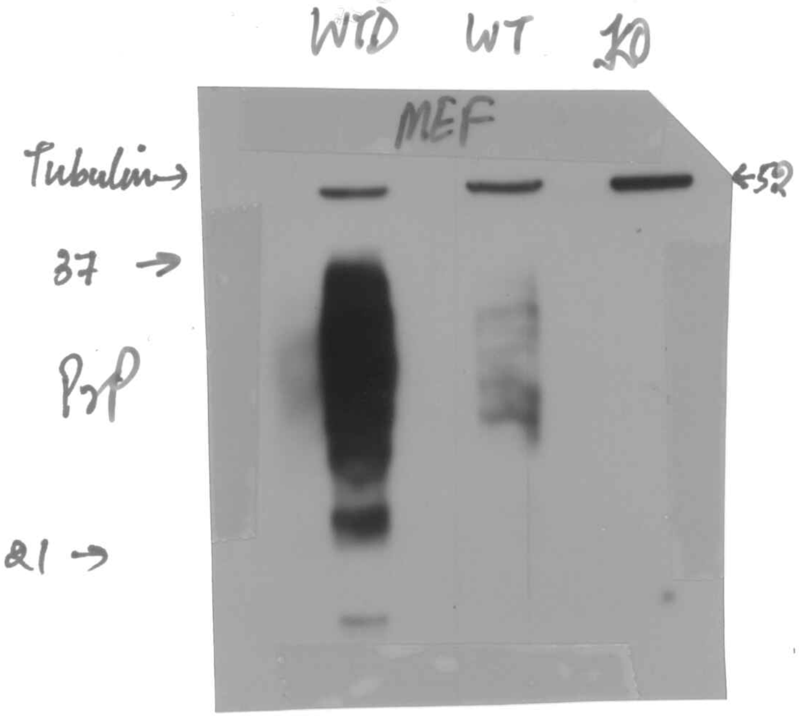

b

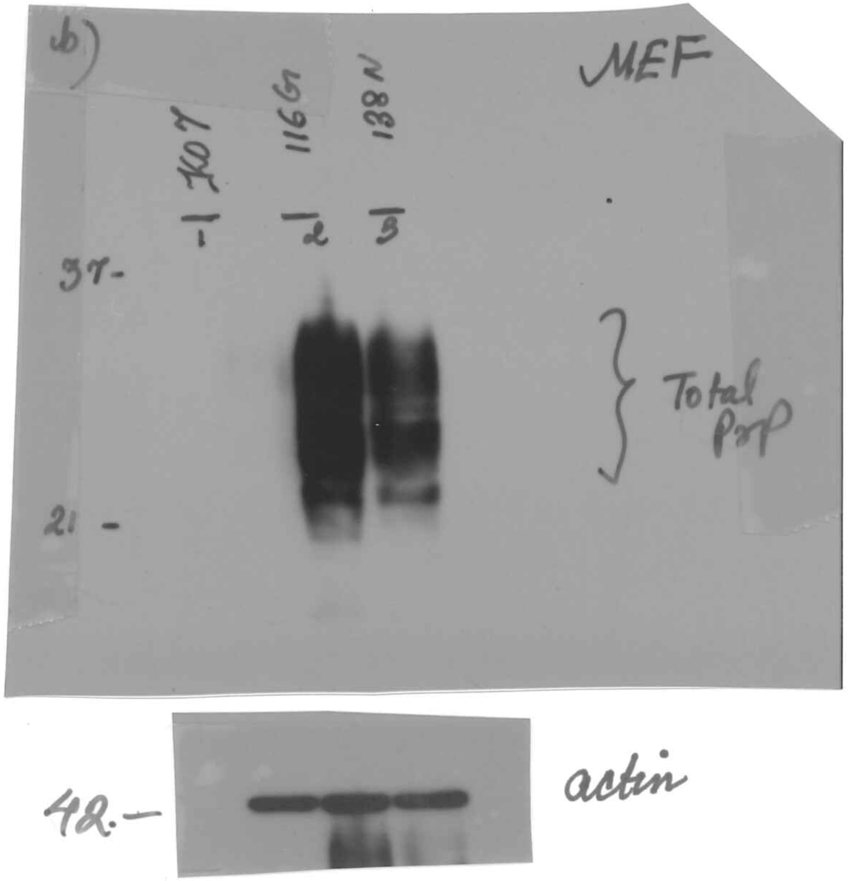

Supplement: Supplementary file 1 — Supplementary data [file 41598_2019_47629_MOESM1_ESM.pdf]
